# Search strategy is regulated by somatostatin signaling and deep brain photoreceptors in zebrafish

**DOI:** 10.1186/s12915-016-0346-2

**Published:** 2017-01-26

**Authors:** Eric J. Horstick, Yared Bayleyen, Jennifer L. Sinclair, Harold A. Burgess

**Affiliations:** Division of Developmental Biology, Eunice Kennedy Shriver National Institute of Child Health and Human Development, Bethesda, MD 20892 USA

**Keywords:** Zebrafish, Search, Motivation, Goal-directed behavior, Non-visual photoreceptor, CRISPR, Orthopedia, Somatostatin, sst1.1, Melanopsin, opn4a

## Abstract

**Background:**

Animals use sensory cues to efficiently locate resources, but when sensory information is insufficient, they may rely on internally coded search strategies. Despite the importance of search behavior, there is limited understanding of the underlying neural mechanisms in vertebrates.

**Results:**

Here, we report that loss of illumination initiates sophisticated light-search behavior in larval zebrafish. Using three-dimensional tracking, we show that at the onset of darkness larvae swim in a helical trajectory that is spatially restricted in the horizontal plane, before gradually transitioning to an outward movement profile. Local and outward swim patterns display characteristic features of area-restricted and roaming search strategies, differentially enhancing phototaxis to nearby and remote sources of light. Retinal signaling is only required to initiate area-restricted search, implying that photoreceptors within the brain drive the transition to the roaming search state. Supporting this, *orthopediaA* mutant larvae manifest impaired transition to roaming search, a phenotype which is recapitulated by loss of the non-visual opsin *opn4a* and somatostatin signaling.

**Conclusion:**

These findings define distinct neuronal pathways for area-restricted and roaming search behaviors and clarify how internal drives promote goal-directed activity.

**Electronic supplementary material:**

The online version of this article (doi:10.1186/s12915-016-0346-2) contains supplementary material, which is available to authorized users.

## Background

Essential resources tend to be clustered, rather than uniformly distributed in the natural environment. Accordingly, animals use a range of behavioral strategies to search for resource rich areas [1]. Search behaviors have been characterized in invertebrate species [2–4], including in *C. elegans*, where detailed underlying neuronal circuits have been described [5–7]. Although sophisticated search behaviors under natural conditions have also been catalogued in vertebrates, surprisingly few studies have exploited vertebrate models to probe genetic factors and neuronal connections that initiate and maintain active search states [8–11].

To date, the powerful repertoire of neurogenetic tools available in zebrafish have been primarily applied to decoding neural mechanisms for acute behavioral responses to sensory stimuli [12, 13]. For instance, larval zebrafish show phototaxis toward restricted areas of illumination in dark environments [14, 15], maneuver within thermal and chemical gradients [16, 17], and actively oppose water currents by swimming against whole-field visual motion. Thus, larvae use sensory information in several modalities to actively navigate within the environment, initiating approach or avoidance behaviors as required. However, much less is known about whether larvae efficiently locate resources that are not in the immediate vicinity. Search behavior requires the maintenance of an internal state that appropriately regulates motor activity, and potentially also modulates sensory thresholds to facilitate the discovery of desirable resources [18, 19]. Short-term internal states such as arousal and hunger, and movement profiles consistent with exploratory behavior are present in larval stage zebrafish [10, 11, 20–22], raising the possibility that autogenic search behavior has also developed.

One promising avenue to investigate search behavior is the hyperactive response of zebrafish larvae to a complete loss of illumination [23–25]. At the onset of darkness, zebrafish larvae become hyperactive for 5–10 min, before settling into a low-activity sleep-like state. The hyperactive state is referred to as the visual motor response, or dark photokinesis [26]. Hyperactivity propels larvae out of dark regions – slower movement in the light causes larvae to aggregate in illuminated regions, an example of classical photokinesis behavior [26, 27].

Here, we investigated whether the locomotor hyperactivity that occurs during dark photokinesis is randomly directed or rather shows spatial patterning that might facilitate light-search behavior. We found that larval swim trajectories during the first few minutes of photokinesis possess characteristic features of a widely utilized animal foraging strategy known as area-restricted search [2, 3, 8, 28]. Area-restricted search is a strategy for temporarily restricting movement to the region where a specific resource was last detected. Typically, area-restricted search is a transient behavior that transitions to a roaming search strategy if the resource is not quickly located [1]. Similarly, we show that after 2–3 min of locally restricted movement, larval swim paths transition to an outward trajectory, consistent with a roaming strategy for light sources that are outside the initial visual range. We demonstrate that the distinct swimming patterns associated with area-restricted and roaming search states respectively increase the ability of larvae to find local and remote light sources. Using high speed recording, we reveal the swim motor patterns associated with area-restricted search and roaming, and show that area-restricted search is generated by utilizing an efficient helical swim pattern composed of same direction turns. Finally, we establish that initiating area-restricted search requires retinal input while the deep brain photoreceptor *opn4a* and *otpa*-specified somatostatin releasing neurons control the transition from intensive local search to a roaming state.

## Results

### Helical swimming follows loss of illumination

**Fig. 1:**
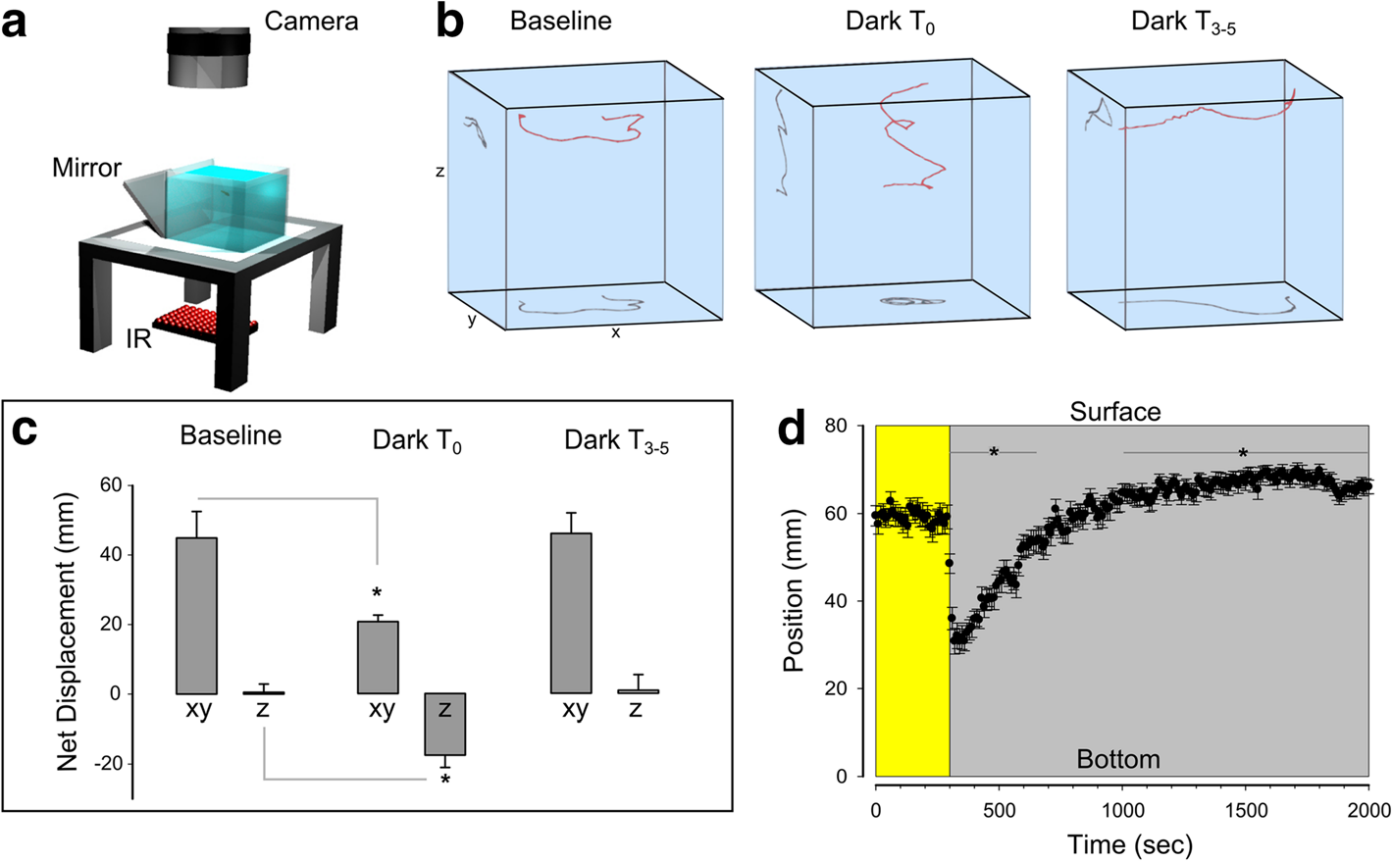
Changes in 3D swim trajectories after loss of illumination. **a** Diagram of 3D recording set up. A mirror was positioned adjacent to arena in order to simultaneously view XY and Z planes. **b** Representative 30-s path trajectories of larvae (*red traces*) during illuminated baseline conditions (*left panel*), immediately (*middle panel*) or 3–5 min after loss of light (*right panel*). Black traces show 2D projections in YZ and XY planes. Chamber size: 85 × 85 × 75 mm. **c** Mean displacement per 30-s recording period in XY and Z axes during baseline swimming (*N* = 10), during the first minute (T_0_; *N* = 25) and 3–5 min after loss of illumination (T_3–5_; *N* = 13). * *P* < 0.05 versus baseline. **d** Mean position of larvae in 80-mm deep chamber during baseline illumination (*yellow background*) and sustained darkness (*grey background*). *N* = 30 groups of five larvae. * *P* < 0.05 versus baseline mean

The visual motor response is a 5- to 10-minute period of hyperactivity that occurs after loss of illumination [24]. In previous studies, the visual motor response has usually been elicited in larvae confined in multi-well plates, precluding the observation of a possible spatial structure in the response. Therefore, to determine whether movement shows a specific spatial pattern or is undirected, we tracked the three-dimensional swim paths of larvae in a large volume chamber (85 × 85 × 75 mm in length, width, and height, respectively) (Fig. 1a). During full-field illumination, locomotor trajectories were primarily in the X–Y plane with relatively little vertical displacement (Fig. 1b, c, baseline). Conversely, during the first minute after loss of illumination, larvae increased downward movement and reduced net displacement in the X–Y plane (Fig. 1b, c, local). These changes reduce net three-dimensional displacement compared to baseline (baseline, 45.5 ± 7.58 mm per 2-minute time interval; T_0_, 30.9 ± 2.73 mm; t-test t_33_ = 2.28, *P* = 0.03). Intriguingly, we noticed that descent trajectories had a helical shape, which was especially apparent when the swim path was projected onto the X–Y plane (Fig. 1b, Dark T_0_). After 3 min in sustained darkness, we observed that movements were similar to baseline, again primarily directed in the horizontal plane (Fig. 1c, Dark T_4_) restoring net three-dimensional displacement (T_4_, 49.0 ± 5.61 mm; t-test vs. baseline, t_21_ = 0.377, *P* = 0.71). Thus, initially after loss of illumination, larvae do not move randomly, but show a strong tendency to slowly swim downwards in the water column, within a spatially restricted region in the horizontal plane.

We next separately analyzed the X–Y planar and vertical components of the response. Taking the vertical component first, we confirmed a previous observation that larvae tend to swim near the top of the water column, and after loss of illumination, swim downwards [26]. However when tested during prolonged loss of illumination, we noted that the ‘diving response’ was transient, lasting for around 1 minute before larvae slowly returned to their baseline position in the water column (time to half recovery = 210 s after light extinction; Fig. 1d). The mean rate of descent following the loss of illumination was 0.75 ± 0.08 mm/s (n = 54), two orders of magnitude slower than the velocity of burst swims (100 mm/s) [29], which are a characteristic feature of escape movements in larvae. Downward swimming is therefore unlikely to be part of a defensive response. Thus, in the vertical dimension, the response to loss of illumination has two phases: a slow downwards swim, followed by a gradual return to the water surface.

### Local movement shows characteristic features of area-restricted search behavior

**Fig. 2:**
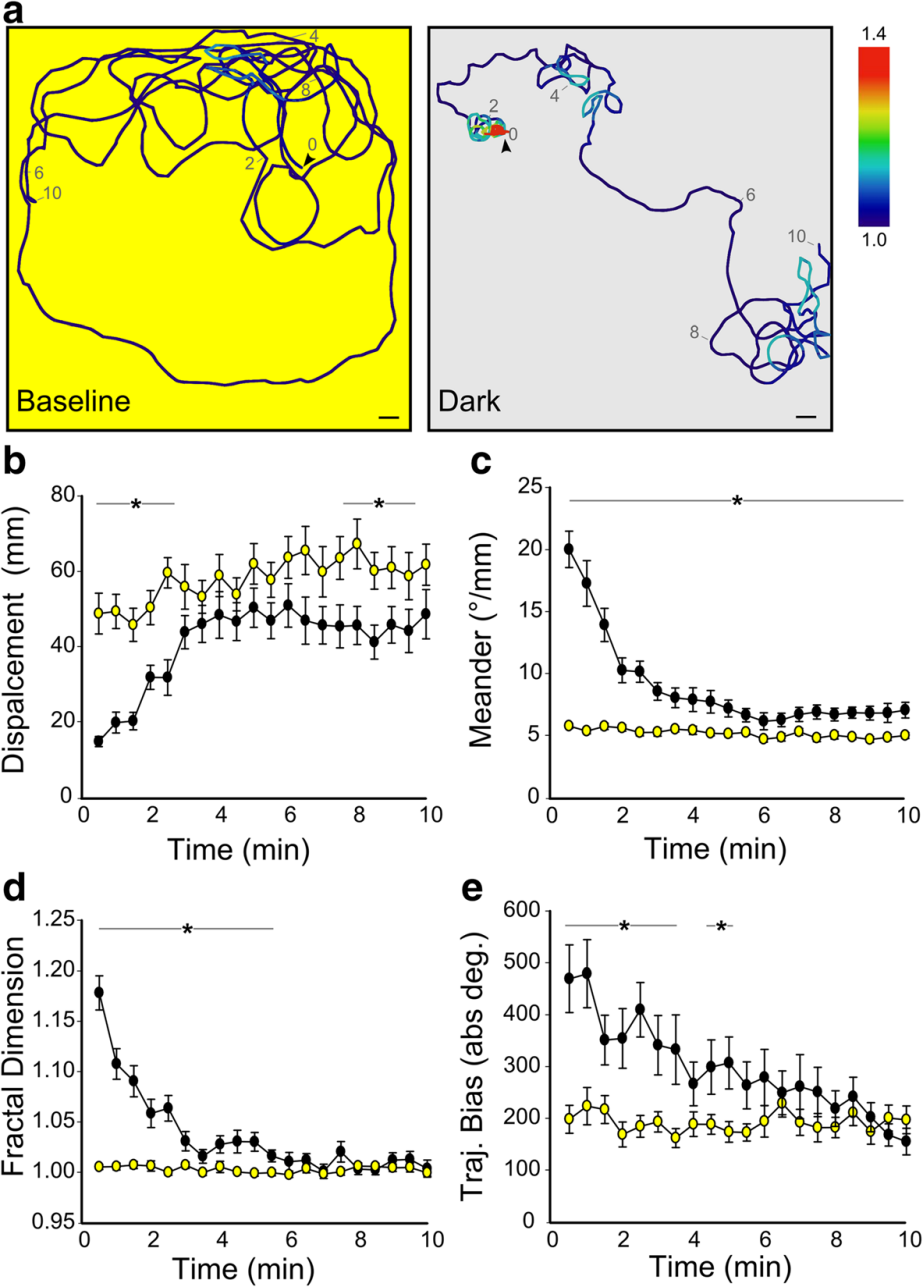
Locally restricted swimming after loss of illumination. **a** Representative path trajectories for a larva during full field illumination (*left panel*) and during the first 10 min after loss of illumination (*right panel*). *Arrowheads* denote starting position and numbers indicate time (min). Color scale indicates fractal dimension in 30 s windows. Main scale bar 10 mm, chamber is 200 × 200 mm. *Arrowhead* length equivalent to larva length. **b**–**e** Displacement, meander, fractal dimension, and trajectory bias for larvae measured during 10 min of full field illumination (*yellow*), followed by 10 min darkness (*black*). *N* = 32. * *P* < 0.05, paired t-test compared to corresponding baseline time-point. See Methods for description of measurements

To study responses in the horizontal plane, we used a large shallow arena (200 × 200 × 5 mm) and tracked individual larvae continuously (10 min baseline, 10 min dark). During baseline conditions, larvae tended to move in long arching paths with relatively low amplitude trajectory changes (Fig. 2a left panel; Additional file 1: Movie 1a). In contrast, after light extinction, larvae initially remained in a restricted area and then gradually transitioned to an outward pattern of swimming similar to baseline (Fig. 2a right panel; Additional file 1: Movie 1b). Accordingly, for the first 3 min after the onset of darkness, travel distance was strongly reduced compared to baseline, confirming that movement was spatially restricted (repeated ANOVA F_1,172_ = 2784.7, *P* < 0.001; Fig. 2b) consistent with reduced displacement in the X–Y plane seen in the three-dimensional environment.

Spatially restricted movement forms part of a behavioral strategy that is used by many animals, known as area-restricted search. Area-restricted search occurs during foraging and mate-search behavior in insects, birds and mammals [2, 8, 28], and during navigation to a shelter or nesting site [30–32]. Characteristic features of area-restricted search are (1) a reduction in travel distance, (2) an increase in movements that cause a change in orientation, (3) an increase in movement path complexity, and (4) a directional bias to movement trajectories [1, 4, 33]. Collectively, these changes allow efficient sampling of the local environment [1]. Similarly, for the first 2–3 min after loss of illumination, in addition to reduced displacement, we found an elevated rate of re-orientation (Meander, Fig. 2c) and greater path complexity (Fractal Dimension, Fig. 2d; see Additional file 2: Figure S1 for illustrative examples).

**Fig. 3:**
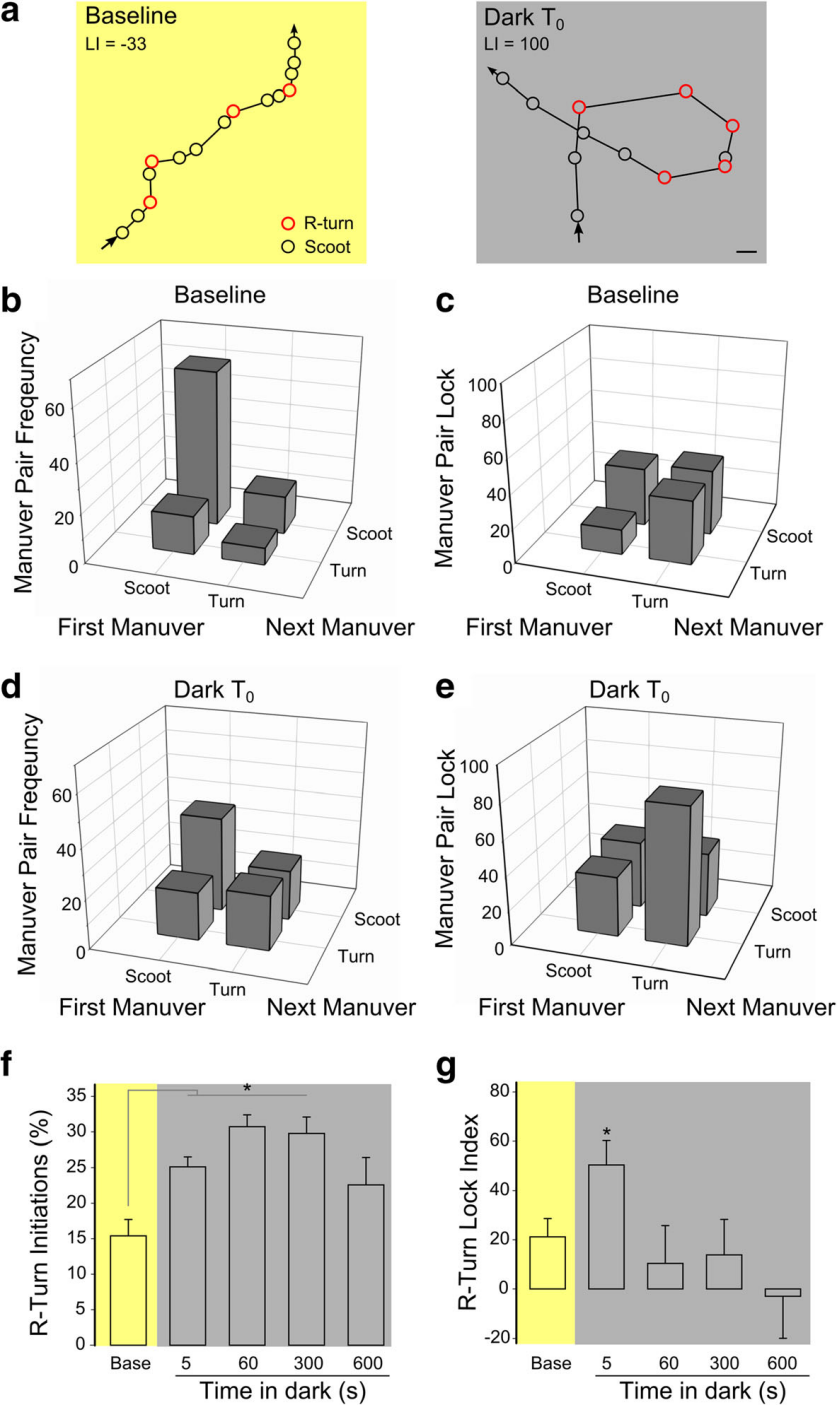
Local search is generated by increased utilization of same direction R-turn maneuvers. **a** Representative 10-s path trajectories of individual larva during baseline *(left, yellow*) and dark (*right, grey*) conditions. *Arrows* indicate path direction of the larva. Scoot (*black circle*) and R-turn (*red circle*) maneuvers are indicated along each path. Lock index (LI) for each recording noted at bottom. Scale bar 2 mm. Frequency (**b**, **d**) and LI (**c**, **e**) of maneuver pairs during full-field illumination (**b**, **c**; baseline) and during the first 10 s after loss of illumination (**c**, **d**; T_0_). Baseline *N* = 39, T_0_ *N* = 55. Maneuver frequency analysis excludes O-bends triggered by the sudden reduction in illumination and infrequent fast burst swims. **c** Baseline maneuver pair LIs are significantly increased over 0 for scoot-scoot (one-sample t-test vs. 0, t_39_ = 8.36, *P* < 0.001), turn-turn (t_24_ = 2.44, *P* = 0.022), and turn-scoot pairs (t_39_ = 4.51, *P* < 0.001). Scoot-turn maneuver pair LI was not significantly different from 0 (t_38_ = 1.01, *P* = 0.32). **f** Frequency of R-turn initiations (percentage of larvae that execute an R-turn per 400 ms analysis window). *N* = 17 groups of 10 larvae each. * *P* < 0.001. **g** LI for sequential R-turns during sustained loss of illumination. *N* = 51 larvae (baseline), 24 (5 s), 22 (60 s), 15 (300 s), 10 (600 s). * *P* < 0.05 compared to baseline time-point

We used two methods to test for directional bias in movement trajectories. First, we noted that, during the initial movement phase, individual trajectories consistently looped either leftward or rightward resulting in a high mean trajectory direction bias (Fig. 2e). Second, we directly measured the frequency of sequential same-direction turning movements. Locomotion in zebrafish larvae consists of discrete maneuvers that are separated by periods of immobility. Under baseline conditions, larvae primarily generate slow swim (scoot) and routine turn (R-turn) maneuver types (Fig. 3a, Baseline), which generate forward propulsion and a change in orientation respectively [29, 34]. Directional bias has been measured using the Lock index (LI), which represents the normalized frequency of sequential maneuvers performed in the same direction [35] (Additional file 3: Figure S2). Consistent with a recent report, we found that under baseline constant illumination, larvae tended to perform sequential swim maneuvers in the same direction, a weak but statistically significant effect [11] (Fig. 3c). For example, during baseline swimming, larvae predominantly executed scoot/scoot maneuver pairs, for which the LI was small, but non-random (LI = 34, one-sample t-test vs. 0, t_39_ = 8.36, *P* < 0.001; Fig. 3b, c, baseline). However, during the local movement phase after loss of illumination, there was a 2.4-fold increase in the frequency of R-turn/R-turn maneuver pairs and a two-fold increase in LI, confirming a strong bias in movement direction (Mann–Whitney U = 11.5, *P* = 0.01; Fig. 3a Dark, d, e). In contrast, during outward swimming, although larvae maintained an elevated rate of R-turn initiation (Fig. 3f), the LI for R-turns declined to baseline levels (Fig. 3g), consistent with the sustained high frequency of re-orienting and loss of directional bias measured using trajectory analysis (Fig. 2c, e). Thus, for the first few minutes after loss of illumination, swim trajectories show a strong directional bias and thus manifest four salient characteristics of area-restricted search behavior.

As for other short-term internal states, search behaviors are typically time-limited and reversible [10]. If a resource is not located within the local environment, a roaming search strategy is often initiated, which is characterized by an outward movement profile [36, 37]. Similarly, spatially restricted movement by larvae persisted only for about 2 min after loss of illumination, after which movement parameters transitioned toward baseline levels. Intriguingly though, two measures, namely displacement and the rate of reorientation, did not match baseline levels even after 10 min (Fig. 2b, c). Principal component analysis confirmed that trajectories in the first minute after loss of illumination were distinct from baseline movements (Additional file 4: Figure S3a), whereas trajectories after 10 min sustained dark did not form a separate group but distributed with either the baseline or first-minute dark response trajectories. Thus, the apparent gradual change in mean trajectory parameters after loss of illumination may in fact be due to changes in the frequency with which larvae switch between discrete episodes of spatially restricted movement, and a movement profile similar to baseline (Additional file 4: Figure S3b–e). Nevertheless, episodes of spatially restricted behavior declined in frequency after loss of illumination, confirming that this response is time-limited (Additional file 4: Figure S3f).

**Fig. 4:**
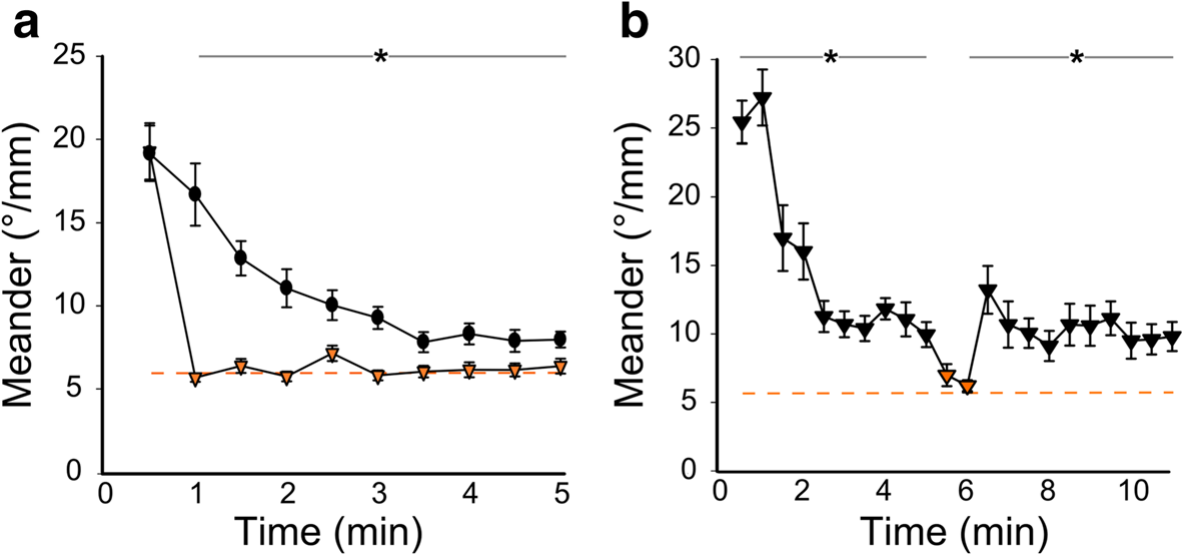
Light reverses changes in locomotor profiles during local and outward swimming. **a** Rate of re-orienting (meander) for larvae measured for 5 min after loss of illumination, either in constant darkness (*black circles*, *N* = 25), or when illumination was restored after 30 s darkness (*orange triangles*, *N* = 22). * *P* < 0.05 for corresponding time-points in sustained dark and after re-activation of the light. **b** Meander for larvae measured for 10 min after loss of illumination, with darkness maintained (*black triangles*) except during 1 min, when the light was re-activated (*orange triangles*). *N* = 19. * *P* < 0.05 compared to constant illumination. *Dashed lines* in (**a**, **b**) indicate mean meander for larvae measured during constant illumination

Behavioral search states are also terminated if a resource is located. To mimic the effect of a successful search, we restored illumination. All locomotor parameters immediately returned to baseline levels when light was resumed either during the local movement phase (two-way ANOVA no main effect of prior dark exposure F_1,135_ = 2.8, *P* = 0.096; Fig. 4a, Additional file 5: Figure S4) or during the outward movement phase (Fig. 4b; t-test, no difference between baseline and light-restored rate of reorientation t_44_ = 1.73, *P* = 0.09). Thus, after loss of illumination, larvae manifest a time-limited and reversible behavior in which they initially swim downwards, while repeatedly executing R-turn maneuvers in a spatially restricted region in the horizontal plane, establishing a helical trajectory. This movement profile is similar to area-restricted search, enabling larvae to intensively survey the three-dimensional environment. If larvae do not locate light, then a new movement profile gradually emerges in which R-turn maneuvers remain elevated but are no longer executed continuously in a single direction, thereby generating outward trajectories. These observations suggest that local movement and outward swim trajectories represent area-restricted and remote light-search behaviors, respectively.

### Local and outward movement patterns differentially facilitate light-search behavior

**Fig. 5:**
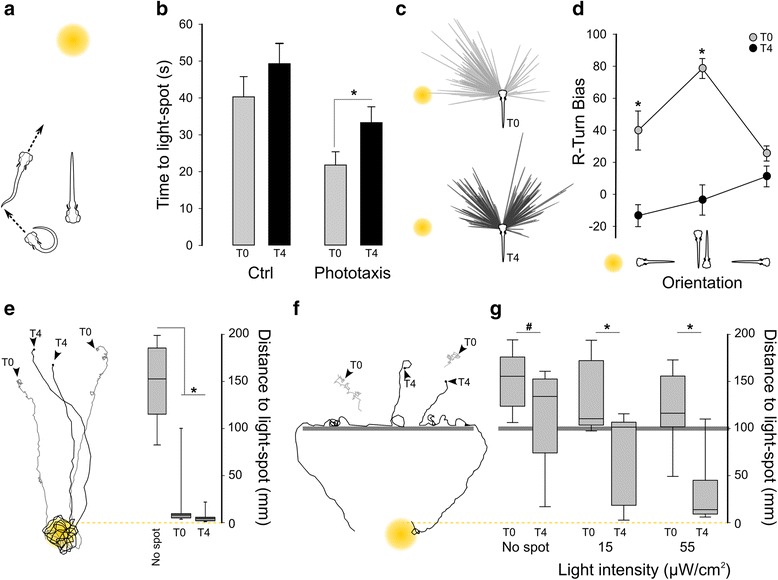
Local and outward movement strategies differentially facilitate locating local and remote light. **a** Schematic of covert phototaxis assay. The light spot (14 mm diameter) was projected 7 mm directly behind a freely swimming larva. We then measured the time for the larva to enter the light spot. **b** Time to light spot perimeter when activated either 2 s (T_0_) or 3–5 min (T_4_) (mean 248 ± 19.6 s, depending on when larvae entered the motion trigger region of interest (ROI)) after loss of full field illumination. Controls: no target light. *N* = 33 (T_0_ control), 40 (T_0_ with spot), 18 (T_4_ control), 20 (T_4_ with spot). **c** Turn maneuver trajectories for larvae oriented with the light spot to their right, when tested 2 s (T_0_) or 3–5 min (T_4_) after loss of full-field illumination. **d** R-turn direction bias during phototaxis relative to a static light spot. Light spot (9 mm diameter, intensity of 20 μW/cm^2^) was illuminated either 2 s (*grey*; *N* = 22 groups of 15 larvae) or 3 min (*black*; *N* = 20 groups) after loss of full field illumination. Orientation of larvae relative to the target spot is indicated. * *P* < 0.05 between T_0_ and T_4_ orientation-matched groups. Bias is the proportion of R-turns directed toward the target spot, normalized between –100 (consistently away from target) to +100 (always toward target). **e** Phototaxis in a large area (200 × 200 mm) using a 55-μW light spot. Representative swim trajectories for larvae tested when the light spot was activated 1 s (T_0_, *grey traces*) or 3–5 min (T_4_, *black traces*) after loss of illumination. *Arrowheads* indicate start positions. Box plot shows closest approach to the light spot for larvae tested 1 s (T_0,_ *grey*) or 3–5 min (mean 247.7 ± 21 s; T_4,_ *black*) after loss of illumination, and for trials where the light spot was not activated (No spot). *N* = 11 larvae (dark, T_0_), 13 (dark, T_4_). **f** Representative swim trajectories for larvae tested when the light spot was activated 1 s (T_0,_ *grey traces*) or 3–5 minute (T_4,_ *black traces*) after loss of illumination. *Arrowheads* indicate start positions. **g** Quantification of concealed target test. Closest approach to the light spot for larvae tested 1 s (T_0,_ *grey*) or 3–5 min (mean 236 ± 13 s; T_4,_ *black*) after loss of illumination with either no target light (No spot), or targets of the indicated intensities. *Horizontal black line* represents position of barrier. *N* = 12 larvae (no spot, T_0_), 12 (no spot, T_4_), 10 (15 μW, T_0_), 11 (15 μW, T_4_), 12 (55 μW, T_0_), and 9 (55 μW, T_4_). # *P* < 0.05, * *P* < 0.001 between T_0_ and T_4_ groups. Experiment was performed as for (**e**) except with an interior barrier

Motivated behaviors such as search-states are goal directed, in that they promote specific objectives [10, 38, 39]. Therefore, to rigorously test whether local and outward movement patterns facilitate light-search behavior, we developed two novel phototaxis assays. First, we established a covert phototaxis assay to test whether spatially restricted movement promotes the discovery of light in the immediate environment. Using real-time tracking we positioned a small (7 mm radius) light spot two body lengths directly behind the larva (Fig. 5a). Because the eyes are positioned laterally and angled slightly forward, the spot was not visible to the larva [40]. We then measured the time taken by larvae to swim into the spot. Larvae reached the spot 34% more quickly when tested during the local movement phase (5 s after loss of full-field illumination), than when tested during the outward movement phase (3–5 min after loss of illumination; U = 238.5, *P* = 0.011; Fig. 5b, Additional file 6: Movies 2a, b). Accelerated phototaxis during the local movement phase could reflect either increased light sensitivity, or earlier light spot detection due to the high rate of re-orientation. To test visual sensitivity, we measured how accurately larvae turned toward a target light within their visual range [15]. Turn movements were strongly biased toward the light during the local movement phase, but not during the outward movement phase (two-way ANOVA, interaction between movement phase and turn bias, F_2,162_ = 11.5, *P* = 0.001; Fig. 5c, d). Thus, during the local movement phase, larvae not only change orientation more frequently than during the outward movement phase, but also show increased responsiveness to a source of illumination.

We next employed a concealed target assay to determine whether outward movement promotes discovery of light that is not in the immediate environment. Here, we used real-time tracking of larval position to project a light spot on the opposite side of a central barrier. The spot was illuminated either 1 s or 3–5 min after loss of full-field illumination to test larvae in the local and outward movement phases, respectively. In trials without a central barrier, larvae rapidly navigated to the light spot regardless of the duration of time spent in the dark (Fig. 5e). Conversely, in the presence of the barrier, larvae only initiated phototaxis if they first swam to the edge of the barrier (Fig. 5f). During control trials (no light spot illuminated) larvae infrequently circumnavigated the barrier (Fig 5g, No spot). Similarly, when the spot was illuminated 1 s after loss of full-field illumination, larvae did not reach the edge of the barrier and therefore did not initiate phototaxis (Fig. 5g; T_0_). In contrast, most larvae tested 3–5 min after loss of full-field illumination swam to the edge of the barrier, then quickly navigated to the light spot (Fig. 5g; T_4_). The difference in final proximity to the light spot was significant (ANOVA, main effect of time in dark F_1,38_ = 36.3, *P* < 0.001), demonstrating that larvae locate remote light sources more rapidly during the outward movement phase.

During the concealed target assay, larvae rarely swam continuously beside the barrier, but often appeared to contact it then move away. This was surprising because several studies have argued that zebrafish larvae perform thigmotaxis (touch-seeking) behavior [34, 41]. However, supporting experiments used an arena with square or concave walls where it is difficult to distinguish true thigmotaxis from mere wall-following due to outward swim trajectories. In fact, under baseline conditions, larvae tended to swim in close contact with the walls of a concave arena but did not show a significant preference to swim in contact with a convex barrier (Additional file 7: Figure S5a–c), indicating that continuous contact with the perimeter of a chamber is a trivial consequence of outward movement trajectories. In contrast, after loss of illumination, larvae tended to avoid the edges of the concave arena (repeated measures ANOVA main effect of illumination on wall proximity F_1,14_ = 49.4, *P* < 0.001; Additional file 7: Figure S5d, e). Local and outward movement phases are thus associated with wall-avoidance behavior.

### Retinal signaling is required to initiate the local movement response

**Fig. 6:**
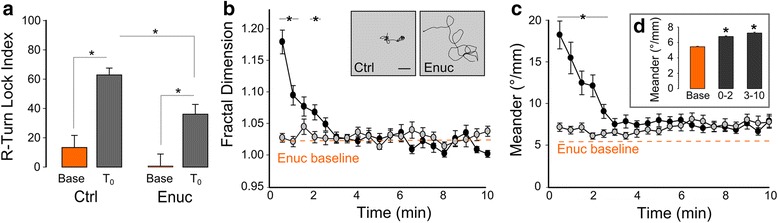
Retinal input is required to initiate local but not outward movement. **a** Lock index for R-turns during baseline and immediately following loss of light (T_0_) during 10 s recordings, for sham operated (ctrl) and enucleated (enuc) larvae. *N* = 41 larvae (ctrl, baseline), 53 (ctrl, T_0_), 44 (enuc, baseline), 59 (enuc, T_0_). * *P* < 0.001. **b**, **c** Fractal dimension (b) and meander (c) of path trajectories during dark response for control (*black circles*, *N* = 29) and enucleated larvae (*grey circles*, *N* = 34). * *P* < 0.05 for control compared to enucleated larvae. *Dashed lines* show mean values for enucleated larvae under full-field illumination. Inset: Representative traces of first 2 min of dark for control (*left*) and enucleated (*right*) larvae. Scale bar 20 mm. **d** Mean meander for enucleated larvae during full-field illumination (base) compared to the first 2 min after loss of illumination (0–2) and from 3–10 min after loss of illumination (3–10). * *P* < 0.001, paired t-test compared to baseline

Larval zebrafish detect and respond to changes in illumination using retinal signaling and intrinsically light sensitive central neurons [25, 26, 42–45]. In particular, both retinal and non-visual photoreceptors drive increased activity after loss of illumination [26]. To assess retinal signaling, we enucleated larvae, then performed kinematic and trajectory analysis. After loss of illumination, the R-turn LI for enucleated larvae increased, confirming a role for non-retinal photoreceptors (Fig. 6a, *Enuc*). However, the LI was 48% reduced compared to intact controls, and trajectory analysis indicated that the increase in locked R-turns was insufficient to drive locally restricted movement (repeated measure ANOVA, no main effect of enucleation on fractal dimension baseline vs. dark, F_1,66_ = 1.06, *P* = 0.31; Fig. 6b, Additional file 8: Figure S6). In contrast, after loss of illumination, the rate of re-orientation for enucleated larvae immediately increased to the level produced by controls during the outward movement phase (Fig. 6c, d). Ablating the pineal complex had no effect on local or outward movement parameters (Additional file 9: Figure S7). Therefore, retinal signaling is required to initiate the local movement phase but extra-retinal signaling mechanisms outside of the pineal are sufficient to drive outward swimming behavior.

### Orthopedia-specified somatostatin-releasing neurons and melanopsin are necessary for search strategy transition

**Fig. 7:**
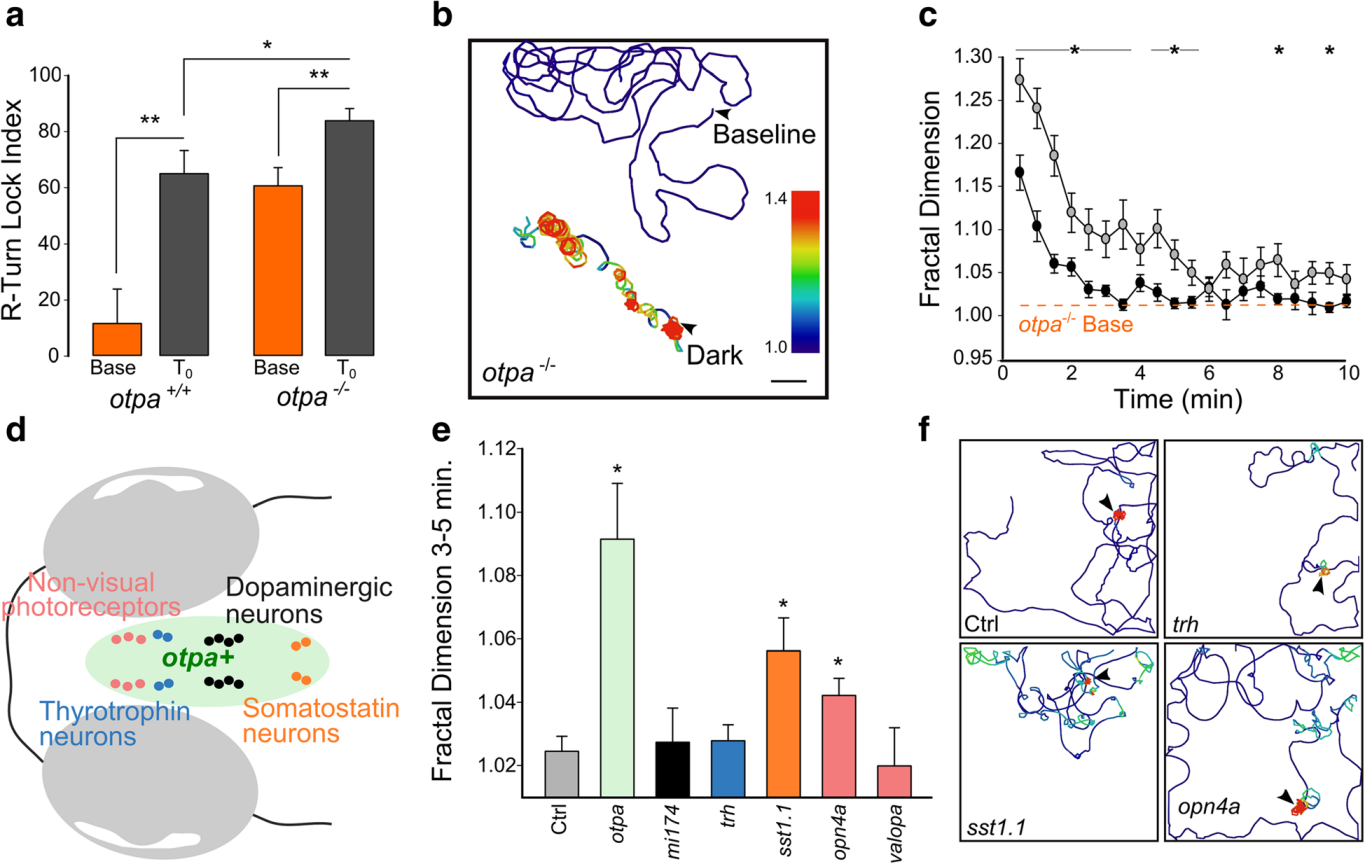
Loss of somatostatinergic neurons and opn4a expressing deep brain photoreceptors potentiates local search. **a** R-turn lock index for *otpa* wildtype siblings and mutants during full-field illumination (base) and immediately following loss of illumination (T_0_). *N* = 25 larvae (sibs, base), 27 (sibs, T_0_), 47 (mutants, base), 38 (mutants, T_0_). **b** Representative path trajectories for an *otpa* mutant larva during 10-min recording periods during baseline (*orange trace*) and after loss of illumination (*black trace*). *Arrowheads* denote starting positions. Chamber: 200 × 200 mm. Scale bar 2 mm. Color represents fractal dimension. **c** Fractal dimension of path trajectories for *otpa* wildtype siblings (*black circles*, *N* = 29) and mutants (*grey circles*, *N* = 31). * *P* < 0.05 for mutants versus siblings. *Dashed line* shows mean for mutants under full-field illumination. **d** Schematic diagram showing neuronal cell types within Orthopedia expression domain. Labeled neuron types correspond to the neuronal markers with reduced expression in the *otpa* mutant background. **e** Mean fractal dimension during 3–5 min following loss of illumination. Control group was injected with sgRNA against GFP in *Tg(vglut2a:EGFP)* transgenic larvae. Controls *N* = 26; *otpa* *N* = 31; *mi174* *N* = 13; *trh* *N* = 37; *sst1.1* *N* = 22; *opn4a* *N* = 36; *valopa* *N* = 6. * *P* < 0.05 for mutant groups versus control group. **f** Representative path trajectories of individual larva for a 10 minute duration following loss of illumination. Top left: CRISPR injected control; Top right: *trh*; Bottom left: *sst1.1*; and Bottom right: *opn4a*. Arrowheads note starting position at time of light extinction. Chamber: 200 × 200 mm. Color represents fractal dimension, with scale as for (**b**)

Hyperactivity after loss of illumination is impaired in the *otpa* mutant, a null for the orthopediaA homeobox transcription factor [26]. After loss of illumination, the R-turn LI in *otpa* mutants was significantly greater than for controls (Fig. 7a) and, accordingly, locally restricted movement was strongly potentiated and persisted for significantly longer, with the movement path complexity remaining elevated throughout the 10-min recording period (two way repeated ANOVA main effect of genotype on fractal dimension F_1,58_ = 17.65, *P* < 0.001; Fig. 7b, c). Thus, in the absence of *otpa* the local movement phase is potentiated and the transition to outward swim trajectories is impaired.

**Fig. 8:**
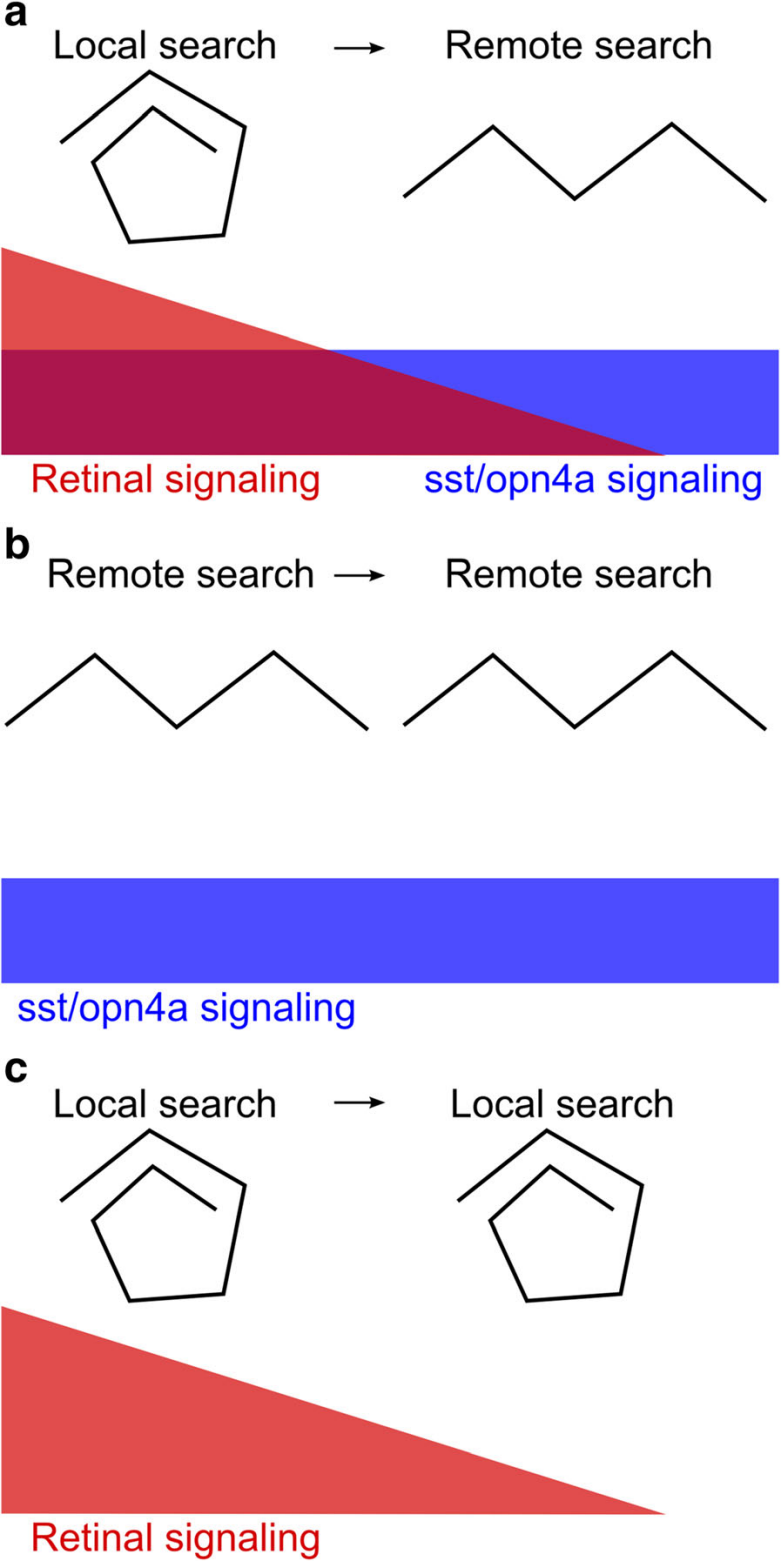
Model for induction of light search behavior by retinal and deep brain photosensory systems. **a** Loss of light detected via the retina drives an initial strong local search (*red*). Simultaneous stimulation of opn4a and sst1.1 signaling drives outward locomotor patterns for remote light sources (*blue*). Local search activity initially masks extended search locomotor features. **b** In enucleated larvae, lack of retinal drive allows remote light-search patterns to emerge immediately after loss of illumination. **c** In the absence of *opn4a* and *sst1.1* to promote outward search in *otpa* mutants, retinal signaling continues to drive local search patterns for a longer period of time, as outward search drive is absent

Enucleated *otpa* mutants did not react to the loss of illumination, confirming that retina- and *otpa*-specified neurons account for all pathways required to initiate local and outward movement profiles (Additional file 10: Figure S8, see legend for statistics). We therefore asked which *otpa*-specified neurons drive transition to the outward movement phase of the response. Otpa specifies multiple neuronal cell types within the larval zebrafish brain, including ventral diencephalon dopaminergic neurons (DA), and thyrotropin- (*trh*) and somatostatin-releasing neurons (*sst1.1*) (Fig. 7d) [46]. We tested the contribution of these cell types using mutant lines, and by directly generating homozygous mutant larvae using CRISPR [47]. For CRISPR mutants, we controlled for off-target cutting by replicating our results using independent guide RNAs against different loci within the target genes. Loss of *sst1.1* recapitulated the delay in swim trajectory transition observed in *otpa*, whereas no significant changes in locomotor output were observed after loss of DA neurons or *trh* neuropeptide (Fig. 7e, f, Additional file 11: Figure S9). *Otpa* mutants also lose light-sensitive melanopsin (opn4a)-expressing neurons in the anterior preoptic region [26]. Accordingly, *opn4a* mutants exhibited a delayed transition to outward swim trajectories, whereas transition was normal after loss of the non-visual opsin *valopa*, which is not specified by *otpa* (Fig. 7e, f, Additional file 11: Figure S9). Thus, among *otpa*-specified neurons, somatostatinergic neurons, and non-visual *opn4a*-expressing photoreceptors drive the transition from local to outward swimming movements (Fig. 8).

## Discussion

We previously proposed that the hyperactivity manifest by larvae in the minutes after loss of illumination promotes aggregation in illuminated regions via photokinesis. Photokinesis is a simple behavioral strategy for navigation that uses rapid non-directional movement in dark areas and slower movement in illuminated regions. However, we now demonstrate that the locomotor activity after loss of illumination is not undirected. Rather, movement trajectories are highly spatially structured and change during the first few minutes in darkness. For the first 1–2 min after loss of illumination, larvae turn intensively to the same side in the horizontal plane, while gradually swimming downwards, resulting in a helical trajectory. After 3 min, larvae continue to execute re-orienting movements at high frequency, but no longer continuously in the same direction; swim trajectories thus propel larvae out of the local environment.

We considered various interpretations of these movement profiles. For instance, light extinction may trigger an aversive or flight response. Indeed, larvae perform a vigorous O-bend response in the first second after loss of illumination [25]; however, because swim travel distance thereafter diminishes below baseline levels, local and outward swim profiles are inconsistent with escape behavior. Alternatively, the high fractal dimension and slow descent during the local movement phase might suggest a disorganized or confused behavior, but this is contraindicated by the highly-accurate light-orientation behavior, efficient phototaxis, stable same-side turning and wall-avoidance that also occur during this period. Finally, these movement profiles are inconsistent with behavioral sleep, which includes reduced sensory thresholds and substantial reductions in locomotor activity [23].

Rather, we interpret the local and outward movement phases as sequential light-seeking responses. Initially, larvae execute an area-restricted light-search behavior, which if unsuccessful, is followed by a remote light-search behavior. Multiple lines of evidence support this view. First, swim trajectories during the local movement phase (reduced travel distance, increased meander, fractal dimension and directionality) are similar to characteristic features of area-restricted search. Similar three-dimensional helical foraging trajectories have been documented in Drosophila and microzooplankton and proposed to yield an optimal search strategy when searching for sparsely distributed food sources [48, 49]. Second, changes to swim profiles were time-limited and rapidly reversed upon restoring illumination, consistent with other motivated states. Third, as for other short-term internal states [20, 21], larvae manifest changes in responsiveness to sensory cues, namely increased sensitivity to a light-spot during the local movement phase, and greater wall avoidance during both local and outward movement phases. Finally, functional tests of light-seeking behavior support this interpretation – larvae more rapidly reached a nearby patch of light during the local movement phase, and conversely located distant illuminated regions only when tested during the outward movement phase. These changes fulfill operational criteria for defining motivated states [10], in that loss of light produces a change in behavior that (1) is maintained internally, (2) is reversible, and (3) facilitates a specific objective. We therefore propose that the swim profiles seen after loss of illumination constitute internally driven search behaviors that enable larvae to efficiently navigate back into illuminated regions. During active phototaxis, movements that re-orient larvae away from a light-spot are rapidly corrected by contraversive turns [15, 35]. However, in the natural environment, a more extensive area-restricted search may be triggered after larvae swim into a small patch of thick foliage or if light is obscured by falling debris. In contrast, sustained remote search activity may be deployed after water currents sweep larvae into large regions of dense undergrowth.

Locomotor responses to loss of illumination are driven by both retinal signaling and *otpa*-specified deep brain photoreceptors [26]. Our results now reveal that retinal signaling and deep brain photoreceptors differentially trigger local and roaming search states; retinal signaling is required for local movement, whereas *otpa*-dependent *sst1.1*-expressing neurons and *opn4a*-expressing deep-brain photoreceptors sustain roaming search behavior. However, search strategy transition was less impaired in *sst1.1* and *opn4a* mutants than in *otpa* mutants. Possible reasons are the presence of supernumerary oxytocin expressing neurons in *otpa* mutants [46], and the presence of 32 nonvisual opsins in the zebrafish [50], raising the potential for uncharacterized additional photoreceptive neurons in the *otpa* expression domain. Nevertheless, our data suggests that after loss of illumination, retinal signaling activates an intense area-restricted search, after which *sst1.1*- and *opn4a*-dependent pathways gradually drive the transition to a roaming search state. Indeed, somatostatin signaling regulates other motivated behaviors, tuning response thresholds during reward [51, 52] and defensive [53] behaviors in mammals. In our current model, *otpa* neurons provide a relatively weak drive, which predominates only when the local search state declines. This idea is supported by the finding that enucleated larvae acutely increase re-orientation rates immediately after the loss of illumination. *Somatostatin* and *opn4a* may therefore continuously drive the roaming search state in darkness, an impulse which is normally masked by retinal-driven local search. Conversely, in the absence of *otpa* circuitry, retinal signaling is unimpeded by remote search drive, prolonging the local response.

Light-search behavior is likely impelled by dwindling nutritional resources. By 6 dpf, yolk supplies are almost depleted and larvae actively pursue prey. Predation requires visual input; larvae are almost completely unable to locate food in darkness [54]. Accordingly, larvae are hard-wired to seek and remain in illuminated regions, demonstrating vigorous phototaxis behavior toward restricted areas of light. During phototaxis, sensory information signals through retinal ON and OFF pathways to trigger forward swims and turns, respectively [15]. Our new results reveal that, in the absence of directional light cues, loss of illumination triggers sequential internally driven light-search strategies involving, first, an area-restricted search for light in the immediate vicinity, followed – if unsuccessful – by a roaming search for light in distant regions.

An unresolved question concerns the “baseline” state of larvae. After 1–2 min of local search, larvae continue executing turn movements at a high rate, but no longer strongly biased to one side, thereby causing outward swimming. Although functionally, outward swimming enabled larvae to locate remote light sources more quickly than during the local movement phase, principle component analysis indicated that outward movement parameters clustered either with local search or baseline swim profiles. Thus, the outward swimming phase, while occasionally interrupted by episodes of local search behavior, showed similar trajectory characteristics to baseline activity under full-field illumination. Nevertheless, wall-avoidance and meander measurements revealed differences between baseline and outward swimming, indicating distinct behavioral states. A strong possibility is that “baseline” behavior under full-field illumination is actually a food-search state; unfed 6 dpf larvae, their yolk supplies exhausted, may already be in a roaming search for food, an idea which is consistent with recent work analyzing movement under constant illumination [11] and changes in sensory responsiveness after feeding [20].

Unexpectedly, net locomotor activity decreased after a loss of illumination. Reduced activity contrasts sharply with many studies on the visual motor response, including from our laboratory, reporting that loss of illumination triggers 5–10 min of hyperactivity [26, 55, 56]. We speculate that chamber size accounts for the discrepancy. In most previous work, movement has been constrained using small wells in which larvae constantly encounter the edge of the arena. However, we found that after loss of illumination, larvae avoid barriers, suggesting that in small arenas such as multi-well plates, where the perimeter is frequently encountered, wall avoidance may trigger increased activity. The rationale for wall avoidance after loss of illumination remains unclear, possibly reflecting a strategy to reduce collisions in darkness, or alternatively facilitating exploration by moving larvae away from barriers that prevent them from detecting distant light sources.

## Conclusion

Larval stage zebrafish larvae maintain distinct behavioral states, including arousal and sleep [10, 20–22]. Our new findings reveal that internal drives in larvae can additionally produce sophisticated motivated behaviors such as search activity. It is likely that larvae exhibit additional types of autogenic search behavior provoked by exposure to, or loss of stimuli in other sensory modalities. The finding that non-retinal photoreception by melanopsin and somatostatin signaling play a selective role in driving remote light-search is consistent with the idea that non-visual photoreceptors are intimately linked to motivational state control [57–59], and provides the opportunity to dissect the ancient evolutionary pathways that allow organisms to adapt to short-term challenges and opportunities in the dynamic natural environment.

## Methods

### Zebrafish husbandry

Zebrafish were maintained on a Tubingen long fin strain background. Larvae were raised at 28 °C in E3 medium, under Light:Dark cycle (14:10 h) and maintained at a maximum density of 30 per 6-cm Petri dish. *otpa* ^*m866*^ homozygous mutant and cousin wildtype pairs (for control experiments) were genotyped as previously described [26]. To test the effect of DA neuron loss we used the previously established *mi174* mutant, which has a global loss or dysfunction of dopaminergic neurons, including Orthopedia-specified DA neurons [60, 61]. Homozygous mutant *mi174* larvae (null for *trpM7*) were identified by the hypopigmentation phenotype and normally pigmented siblings from the same clutches were used for controls [62] (kind gift from Jim Hudspeth lab). Enucleations were performed on 4 dpf larvae that were anaesthetized in Evans solution with tricaine. Using a Sylgard-coated dish with a groove to steady the larva, both eyes were removed with sharp forceps. After enucleation, larvae were allowed to recover in fresh Evans solution (134 mM NaCl, 2.9 mM KCl, 2.1 mM CaCl_2_, 1.2 mM MgCl_2_, 10 mM glucose and 10 mM Hepes pH 7.8). After approximately 24 h larvae were transferred to E3 media. Control larvae were treated with tricaine and incubated in Evans solution in parallel with enucleated larvae. Enucleation produced characteristic melanophore expansion and a complete loss of high angular O-bend responses in response to a dark flash [63], confirming that larvae were functionally blind (not shown). All in vivo experimental protocols were approved by the NICHD animal care and use committee.

### Behavioral testing

Tests were performed on larvae at 6 or 7 dpf. Behavioral assays were monitored using infrared illumination (CMVision Supplies, 850 nm) positioned below the chamber, with IR-longpass filters on the camera lens to exclude visible light. For white-light illumination we used LEDs positioned above the chamber unless otherwise specified. Prior to behavioral recording larvae were adapted for a minimum of 30 min to the same light intensity as in the testing arena. Light intensities were measured using a radiometer (International Light Technologies). Testing arenas were maintained at 28 °C. Image acquisition and illumination were controlled using DAQtimer event control software [63]. We added new motion trigger code to DAQtimer to detect when a larva entered a specified ROI. For kinematic analysis, we recorded video at 1000 frames per s (fps) using a Fastcam 1024 (Tech Imaging). Tracking was performed offline using Flote tracking software, which also measured swim kinematics and classified locomotor maneuvers [63]. For trajectory analysis (all recordings at less than 1000 fps) images were captured with a μEye IDS-1545LE-M CMOS camera (1^st^ Vision, Andover, MA), tracked in real-time using DAQtimer [21], and analyzed offline using custom scripts written in IDL (Exelis, Boulder, CO).

### 3D trajectory analysis

Single larvae were placed in an 85 × 85 × 85 mm clear acrylic chamber filled to a depth of 75 mm, illuminated at 50 μW/cm^2^. Images were captured at 20 Hz with the recording window adjusted to simultaneously record the X–Y plane and the vertical Z position of the larvae using a mirror placed adjacent to the chamber at a 45° angle. Each larva was adapted to the arena for at least 5 min and recordings were started when the larva was centrally positioned in the arena. Tracking was performed offline and horizontal and vertical displacement analyzed in 30-s time windows.

### Dive analysis

Groups of five larvae were placed in a 40 × 45 × 100 mm (length × width × height) clear acrylic chamber filled to a height of 80 mm, illuminated at 200 μW/cm^2^. Larvae were adapted to the arena for 10 min before testing. Recordings were captured at 2 Hz and included 300 s of full-field illumination (baseline) followed by 1700 s in darkness. The mean position of the larvae was analyzed in 2.5 s bins. We measured descent speed using individual larvae in the same container during the period from 1.6 to 6.0 s after loss of illumination to capture the linear portion of the response (i.e. after initiation of the downward swim but before larvae reached the bottom of the arena).

### 2D trajectory analysis

Single larvae were placed in a 200 × 200 mm chamber (5 mm depth, illumination 50 μW/cm^2^). After a 10 min adaptation period, recordings were initiated once the larva entered a 120 × 120 mm motion trigger ROI in the center of the arena, after which the larva was tracked at 10 Hz under constant illumination for 10 min. The motion trigger ROI was then restored. Once the trigger was again activated, illumination was turned off and the larva tracked for a second 10-min period. For each larva we then used 30-s track segments to measure displacement, distance, meander, fractal dimension, and trajectory bias. Displacement: the straight-line distance between the starting and ending point for each 30 s interval. Distance: total travel distance, calculated as the total of the straight-line distances covered in each of the thirty 1 s line segments. Meander: a measure of the rate of reorientation [4], calculated using the total of the absolute values of angles formed by all trajectory changes during the 30 s interval, divided by the distance. Fractal dimension: a measure of path complexity [64] frequently used in studies of search behavior [28, 49, 65], which we implemented using the box-counting method [66]. In brief, the fractal dimension was estimated as the slope of the line fitting the log ratio of the number of box units that cover part of the trajectory versus box size. Trajectory bias: a measure of the directional (left or right) bias of the trajectory, calculated using the absolute value of the total of the angles formed by all trajectory changes during the 30-s interval. Leftward and rightward trajectory changes produced negative and positive angles, respectively; thus, these changes summed to near zero when larvae swam in predominantly straight lines. As tracking was not reliable when larvae were adjacent to the wall of this arena, we excluded segments that included less than 10 s of data. Principal component analysis was performed on measures of displacement, meander, fractal dimension, and trajectory bias after standardization to unit variance and mean-subtraction.

### Covert phototaxis assay

To measure how quickly larvae swam to a nearby light source, we used a 58 × 58 mm chamber (depth 5 mm), illuminated overhead at 50 μW/cm^2^. Single larvae were placed in the arena, adapted for 2 min and tracked in real-time. After the larva swam into a 20 × 20 mm motion trigger ROI in the center of the arena, full-field illumination was extinguished. After either 3 s or 3 min in darkness, we established a second motion trigger in the center of the arena. Once this was triggered we used the position and orientation of the larva (from real-time tracking) to project a stationary 14-mm diameter light spot of 23 μW/cm^2^ intensity at a position 7 mm directly behind the tail of the fish (AAXA P2 Pico Projector). We recorded the response of the larva, and the position of the light-spot using a second camera with an IR-blocking filter at 60 Hz, then measured offline the time for the larva to enter the perimeter of the spot. During “no target” control experiments the light spot was flashed on for 50 ms, then extinguished, so that we could calculate the time taken for the larva to swim into an equivalent target region by chance. Larvae reached the target spot significantly more quickly when it remained illuminated, confirming that efficient phototaxis occurred (ctrl vs. phototaxis: for T_0_, t_56.6_ = 2.8, *P* = 0.007; for T_4_, t_36_ = 2.5, *P* = 0.016).

### Concealed target assay

The efficiency of locating a light source that was not immediately visible was tested using a 200 × 200 mm chamber (5 mm depth, illumination 50 μW/cm^2^). Two LEDs were positioned beneath the chamber on opposite sides, each with an iris to produce a 25-mm light spot on a diffuser immediately below the chamber. We first confirmed that larvae performed robust phototaxis to a light spot illuminated on the opposite side of the chamber (Additional file 4: Figure S3). Then, to prevent larvae from directly detecting the position of a light spot on the opposite side, we added a 140-mm opaque barrier into the middle of the arena. After 10 min adaptation to the arena, we established a rectangular motion trigger ROI in the center of the arena (20 mm away from the ends of the barrier, encompassing regions on both sides of the barrier). After the trigger was activated, full-field illumination was turned off. Next, either 1 s or 3–5 min after the onset of darkness, a light-spot was activated on the side of the barrier opposite to the position of the larva. The larva was then tracked for a 2-min period during which we measured the closest position of each larva to the center of the light spot.

### Turn bias assay

Groups of 15 larvae were recorded simultaneously in a 90-mm diameter arena (depth 4 mm, illumination 50 μW/cm^2^). After 5 min adaptation, we extinguished full-field illumination. Then, after either 2 s or 3 min, we induced phototaxis using a 9 mm diameter 20 μW/cm^2^ light spot illuminated on a diffuser below one side of the arena and recorded responses for 12 s with a high speed camera. Turn bias was the tendency of R-turn maneuvers to be made toward the light spot as previously described [15]. Briefly, an index of –100 means all turns are directly away from the spot, and +100 means turns are executed toward the spot. For analysis, larvae were binned based on their orientation relative to the spot.

### Kinematic analysis

To determine maneuver pair frequency and LI we used a 40 × 40 mm chamber, illuminated at 200 μW/cm^2^. Single larvae were acclimated in the chamber for 2 min before a 15 × 15 mm motion trigger ROI in the center of the chamber was established. After the trigger was activated, we collected 10 s of data using a high speed camera, either maintaining illumination (baseline) or immediately after turning the light off. Larvae were tracked using Flote, then custom scripts were used to calculate the frequency and LI for each maneuver pair. To determine the initiation frequency of R-turns under sustained loss of illumination, experimental parameters were as described above with the following changes. Larvae were recorded in groups of five and acclimated for a period of 30 min after being placed into the recording arena. Initiation frequency is per 400 ms analysis window. To measure the R-turn LI during sustained darkness, single larva were recorded at the specified intervals over a 10-min time-period. The LI is the percentage of sequential maneuvers that are performed in the same direction, normalized between –100 and +100, such that random turning has a LI of 0, whereas for constant same-side turning the LI is +100 (as illustrated in Additional file 3: Figure S2). Only trials in which a larva executed at least three R-turns were used when calculating the LI.

### Gene mutations

Cloning free CRISPRs were designed and synthesized using methods as described [47]. sgRNAs were designed using CRISPRscan [67]. CRISPR sgRNA (120 pg), cas9 RNA (300 pg), and fluorescent protein RNA (30 pg) were co-injected into single cell wildtype embryos. Fluorescent protein RNA (either RFP or GFP) was used as a marker for successful injection and used to sort embryos for behavioral testing. Two-dimensional trajectory behavior and analysis, described above, was performed on successfully injected larvae. CRISPR efficacy was determined for every larva tested using CRISPR-STAT fluorescent PCR (Additional file 12: Figure S10) using an ABI 3100 Genetic Analyzer Avant [68]. Additional file 13: Table S1 lists guide RNA sequences and genotyping primers. Custom software was used to measure percent of wildtype gene target degradation for semi-quantitative analysis of knockdown efficiency. Only larvae with bi-allelic conversion of the wildtype gene products were included for quantification – a maximum of 5% detectable wildtype PCR product was used as a threshold for inclusion into the data set. For control experiments, we injected a guide RNA targeting EGFP into in-crosses of a transgenic zebrafish line *Tg(vglut2a:EGFP)* which was derived from *nns14Tg* by Cre injection [69]. Injected control larvae were processed as for experimental groups after assessing loss of EGFP PCR product.

### Pineal ablation

Neuronal ablation was performed as previously described [70]. Briefly, 1 dpf larvae from in-crosses of *y227Tg*, a line with pineal-specific expression of nitroreductase-tagged mCherry, were treated with 10 mM metronidazole in E3 media for 48 h with drug replacement after 24 h. After treatment, larvae were returned to fresh E3 media. Ablation efficacy was assessed by screening for mCherry fluorescence. Controls were wildtype siblings, also treated with 10 mM metronidazole.

### Statistical analysis

Statistical analysis was performed using SPSS (IBM, Armonk, NY), IDL and Gnumeric (Available from: http://www.gnumeric.org/). Graphs show mean and standard error of the mean unless otherwise specified. Normality of data sets was determined using the Shapiro–Wilk test in SPSS. Significance for normally distributed data sets was calculated using the two-tailed Student t-test. For data not normally distributed, the Mann–Whitney U test was applied.

## Additional files

Additional file 1: Movie 1a, b. Representative path trajectory of a larva during (a) baseline illumination and (b) after loss of illumination. Movie duration 10 min. (ZIP 2209 kb)
Additional file 2: Figure S1. Fractal dimension measurement of path complexity. Illustrative examples of fractal dimension values for 30 s duration movement paths of increasing complexity. Start position of each path is indicated (black circle). (TIF 503 kb)
Additional file 3: Figure S2. Schematic of lock index measure. Left: random turning produces a Lock index of zero. Right: continuous turning in one direction gives a Lock index of 100. (TIF 352 kb)
Additional file 4: Figure S3. Periods of area-restricted movement re-occur sporadically during prolonged dark. (a) First three principal components of trajectory measures, which together account for 95.5% of the variance. Small spheres represent values for 1 minute periods taken from baseline (orange), first minute after loss of illumination (grey) and tenth minute after loss of illumination (blue). (b) Fractal dimension for 10 larva during baseline (orange) and paired dark response (grey). (c) Distribution of fractal dimension values for 30 s windows during baseline (orange) and dark response (grey). *N* = 32 larvae, 40 time-points each. A threshold of 1.10 for local-search behavior (arrow) is exceeded by 0.5% of traces during baseline recordings, and marks an inflection point in the distribution of values during the dark response. (d) Traces from two larvae over a 10-minute recording after loss of illumination. Boxes indicate period where the fractal dimension of the trajectory exceeded 1.10 during the outward swimming phase of the response. Arrowheads indicate starting locations. (e) Fractal dimension from 3 to 10 min after loss of illumination for the larvae indicated in (b). Red dotted line indicates the threshold value for local-search like behavior. (f) Frequency of ARS in 30 s time bins, as defined by episodes exceeding the fractal dimension threshold of 1.1. Percent shows proportion of larvae (*N* = 34) manifesting ARS behavior per time-point. (TIF 2394 kb)
Additional file 5: Figure S4. Light restores baseline trajectory profiles. Fractal dimension (a) and trajectory bias (b) after loss of illumination, either with constant dark (black circles, *N* = 25) or when full-field illumination was restored after 30 s of dark (orange triangles, *N* = 22). Dotted line indicates mean for larvae tested under constant illumination (*N* = 25). * *P* < 0.05 for corresponding time-points between larvae in constant dark versus larvae with illumination restored. (TIF 716 kb)
Additional file 6: Movies 2a, b. Representative response of a larva in the local search assay after (a) a 3 s delay following loss of illumination, and (b) after a 3 minute delay following loss of illumination. (ZIP 6033 kb)
Additional file 7: Figure S5. Wall-avoidance behavior after loss of illumination. Illustrative path trajectories for larvae over 10 min, inside a transparent interior barrier (diameter 85 mm) within the recording chamber (a) or outside the barrier (b) under baseline conditions. (c) Time spent within concentric rings of equal area (1000 mm^2^) progressively further from the interior barrier during 10 min recording as in (b). Differences between time spent in the four rings are not significant (repeated measures ANOVA F_3,39_ = 0.19, *P* = 0.90), indicating that larvae do not show preferential swimming in proximity to a convex wall; *N* = 14 larvae. (d) Representative 2 min trajectories for larva inside the interior barrier during baseline (orange), or 8 min after loss of illumination (black). (e) Quantification of (d) – proportion of time spent by larvae within 3 mm of the wall of the chamber during baseline and at the indicated time-points after loss of illumination; N = 15. * *P* < 0.05. (TIF 1499 kb)
Additional file 8: Figure S6. Enucleated larva path trajectories. Representative 10 min trajectory for a control (a, b) and an enucleated larva (c, d) during baseline full-field illumination (a, c) and after loss of illumination (b, d). Arrow indicates starting position of larva at the beginning of the recording. Chamber: 200 × 200 mm. Color scale represents fractal dimension along path trajectory. (e–f) R-turn and slow-swim initiations were not significantly different between control and enucleated larvae after loss of illumination, demonstrating that enucleation did not broadly perturb behavior (independent sample t-test: R-turn t(121) = 1.5, *P* = 0.129; Scoot t(121) = 0.139, *P* = 0.889). (TIF 1543 kb)
Additional file 9: Figure S7. Response of pineal ablated larvae to loss of illumination. (a) Fractal dimension and (b) meander after loss of illumination in pineal ablated *y227Tg* larvae (grey, *N* = 31) and metronidazole treated non-transgenic siblings (black, *N* = 16). Dashed line shows mean for ablated larvae under full-field illumination. (TIF 1041 kb)
Additional file 10: Figure S8. Response of enucleated *otpa* mutant larvae to loss of illumination. Displacement (a), path complexity (fractal dimension, b), rate of re-orientation (meander, c), and trajectory bias (d) for enucleated *otpa* homozygous mutant larvae, recorded for 10 min under full-field illumination (yellow) or following loss of illumination (black); *N* = 22. Repeated measures ANOVA showed no significant main effect of enucleation on movement parameters (fractal dimension: F_1,38_ = 0.292, *P* = 0.59; displacement: F_1,40_ = 0.66, *P* = 0.42; meander: F_1,38_ = 2.28, *P* = 0.14; traj. bias: F_1,38_ = 0.087, *P* = 0.77). (TIF 1296 kb)
Additional file 11: Figure S9. Confirmation of sst1.1 and opn4a mutant phenotypes using an independent guide RNA. Path complexity for *sst1.1* and *opn4a* mutants generated using independent sgRNAs, targeting different genomic sites than for the experiments in Fig. 7. Quantification is as for Fig. 7e. * *P* < 0.05 for mutants versus controls. Controls *N* = 26; *sst1.1* T2 *N* = 26; *opn4a* T2 *N* = 43. (TIF 494 kb)
Additional file 12: Figure S10. Screening mutant phenotypes by CRISPR-mediated biallelic gene mutation. Representative fluorescent PCR results for amplification around the CRISPR target site in the *opn4a* gene in: (a) uninjected embryo showing wildtype 144 bp product from opn4a primers. (b) Embryo injected with cas9 and sgRNA against opn4a showing a residual 144 bp peak indicating incomplete mutation of opn4a. Larvae with similar genotypes were excluded from analysis. (c) Injected embryo showing complete loss of the wildtype PCR product, indicating successful biallelic mutation. Peak sizes labeled below each trace. (TIF 1371 kb)
Additional file 13: Table S1. CRISPR design and genotyping. (XLS 19 kb)
