# Supplementary figures and images for: Search strategy is regulated by somatostatin signaling and deep brain photoreceptors in zebrafish
Source: BMC Biol. 2017 Jan 26;15:4. doi: 10.1186/s12915-016-0346-2 (PMC5267475; doi:10.1186/s12915-016-0346-2)

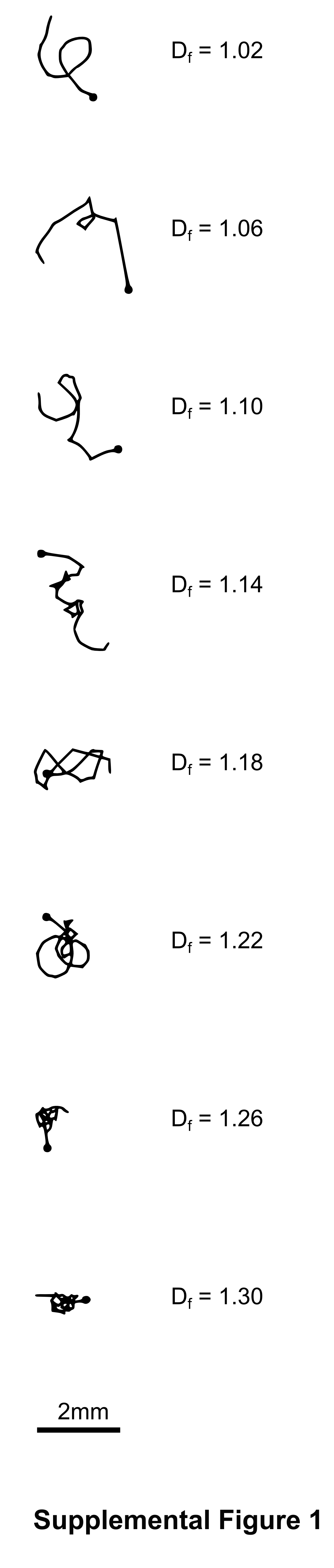

Supplement: Additional file 2: Figure S1. — Fractal dimension measurement of path complexity. Illustrative examples of fractal dimension values for 30 s duration movement paths of increasing complexity. Start position of each path is indicated (black circle). (TIF 503 kb) [file 12915_2016_346_MOESM2_ESM.tif]

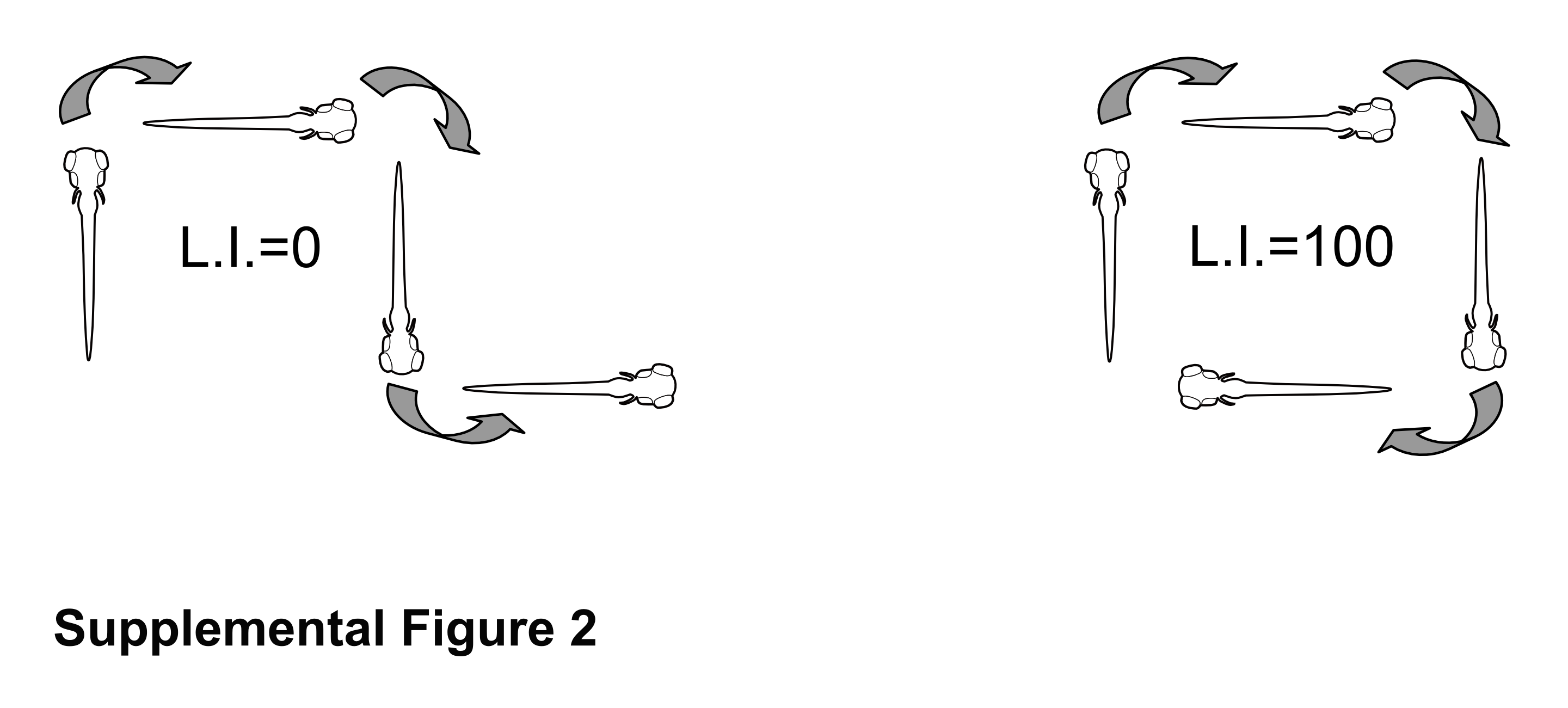

Supplement: Additional file 3: Figure S2. — Schematic of lock index measure. Left: random turning produces a Lock index of zero. Right: continuous turning in one direction gives a Lock index of 100. (TIF 352 kb) [file 12915_2016_346_MOESM3_ESM.tif]

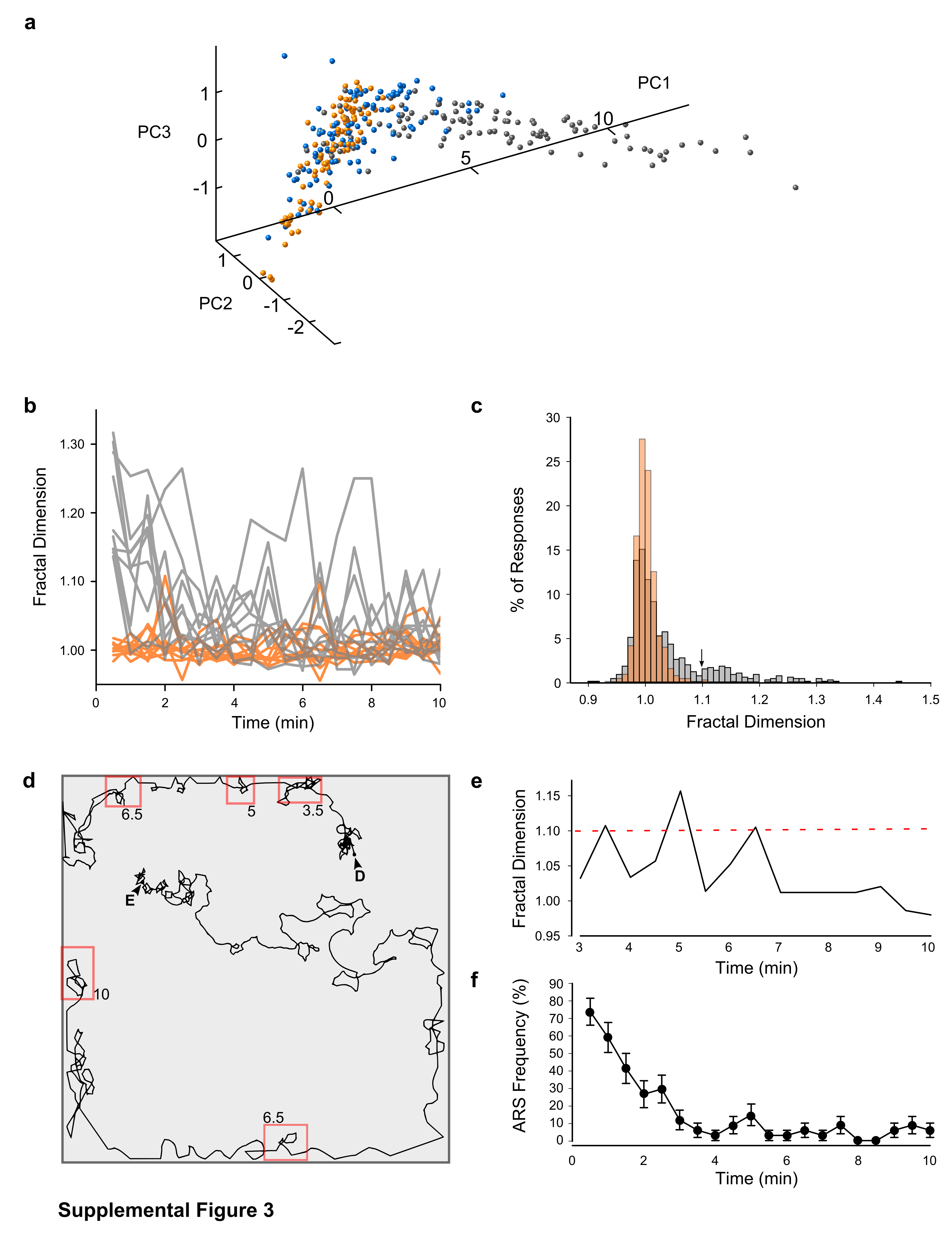

Supplement: Additional file 4: Figure S3. — Periods of area-restricted movement re-occur sporadically during prolonged dark. (a) First three principal components of trajectory measures, which together account for 95.5% of the variance. Small spheres represent values for 1 minute periods taken from baseline (orange), first minute after loss of illumination (grey) and tenth minute after loss of illumination (blue). (b) Fractal dimension for 10 larva during baseline (orange) and paired dark response (grey). (c) Distribution of fractal dimension values for 30 s windows during baseline (orange) and dark response (grey). N = 32 larvae, 40 time-points each. A threshold of 1.10 for local-search behavior (arrow) is exceeded by 0.5% of traces during baseline recordings, and marks an inflection point in the distribution of values during the dark response. (d) Traces from two larvae over a 10-minute recording after loss of illumination. Boxes indicate period where the fractal dimension of the trajectory exceeded 1.10 during the outward swimming phase of the response. Arrowheads indicate starting locations. (e) Fractal dimension from 3 to 10 min after loss of illumination for the larvae indicated in (b). Red dotted line indicates the threshold value for local-search like behavior. (f) Frequency of ARS in 30 s time bins, as defined by episodes exceeding the fractal dimension threshold of 1.1. Percent shows proportion of larvae (N = 34) manifesting ARS behavior per time-point. (TIF 2394 kb) [file 12915_2016_346_MOESM4_ESM.tif]

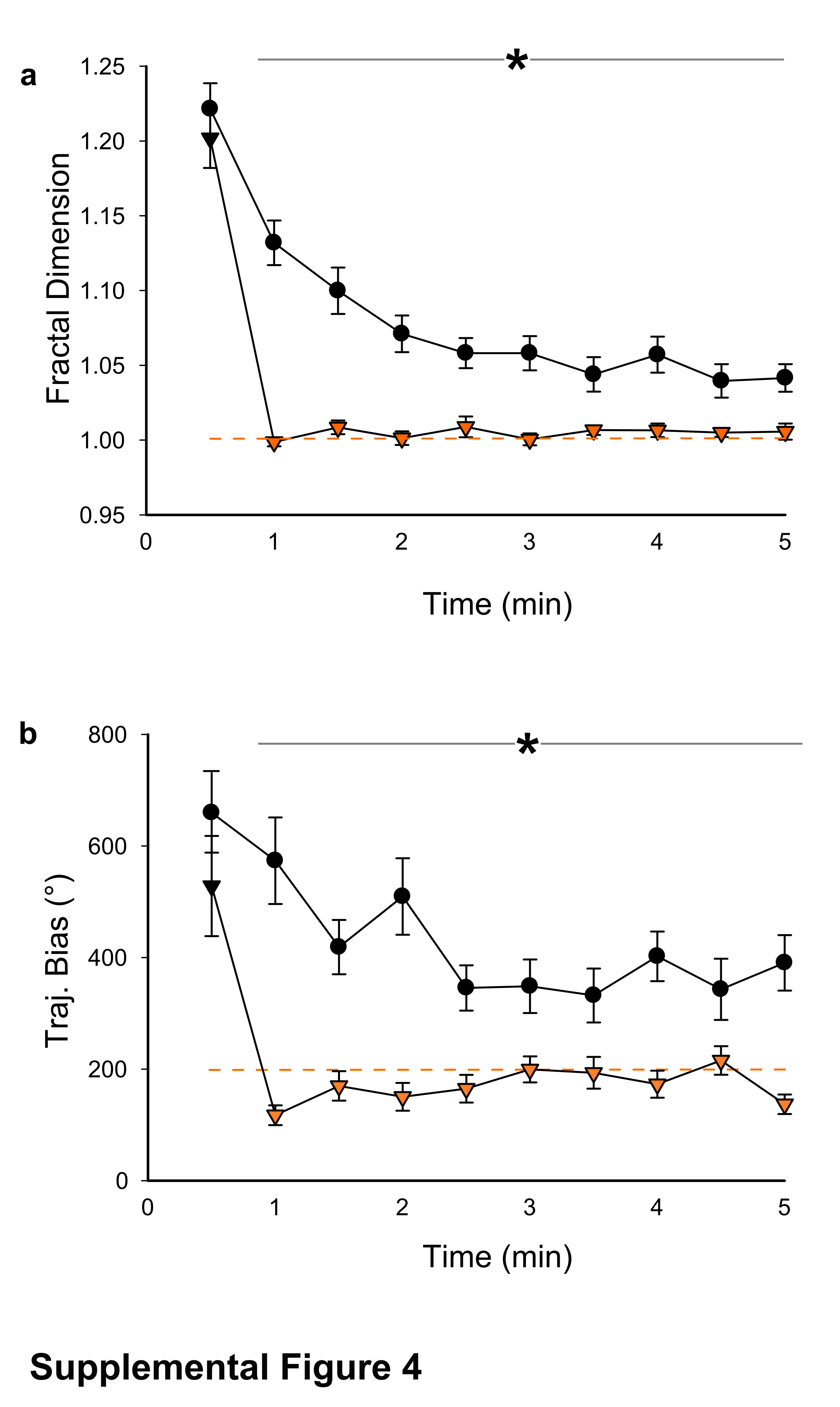

Supplement: Additional file 5: Figure S4. — Light restores baseline trajectory profiles. Fractal dimension (a) and trajectory bias (b) after loss of illumination, either with constant dark (black circles, N = 25) or when full-field illumination was restored after 30 s of dark (orange triangles, N = 22). Dotted line indicates mean for larvae tested under constant illumination (N = 25). * P < 0.05 for corresponding time-points between larvae in constant dark versus larvae with illumination restored. (TIF 716 kb) [file 12915_2016_346_MOESM5_ESM.tif]

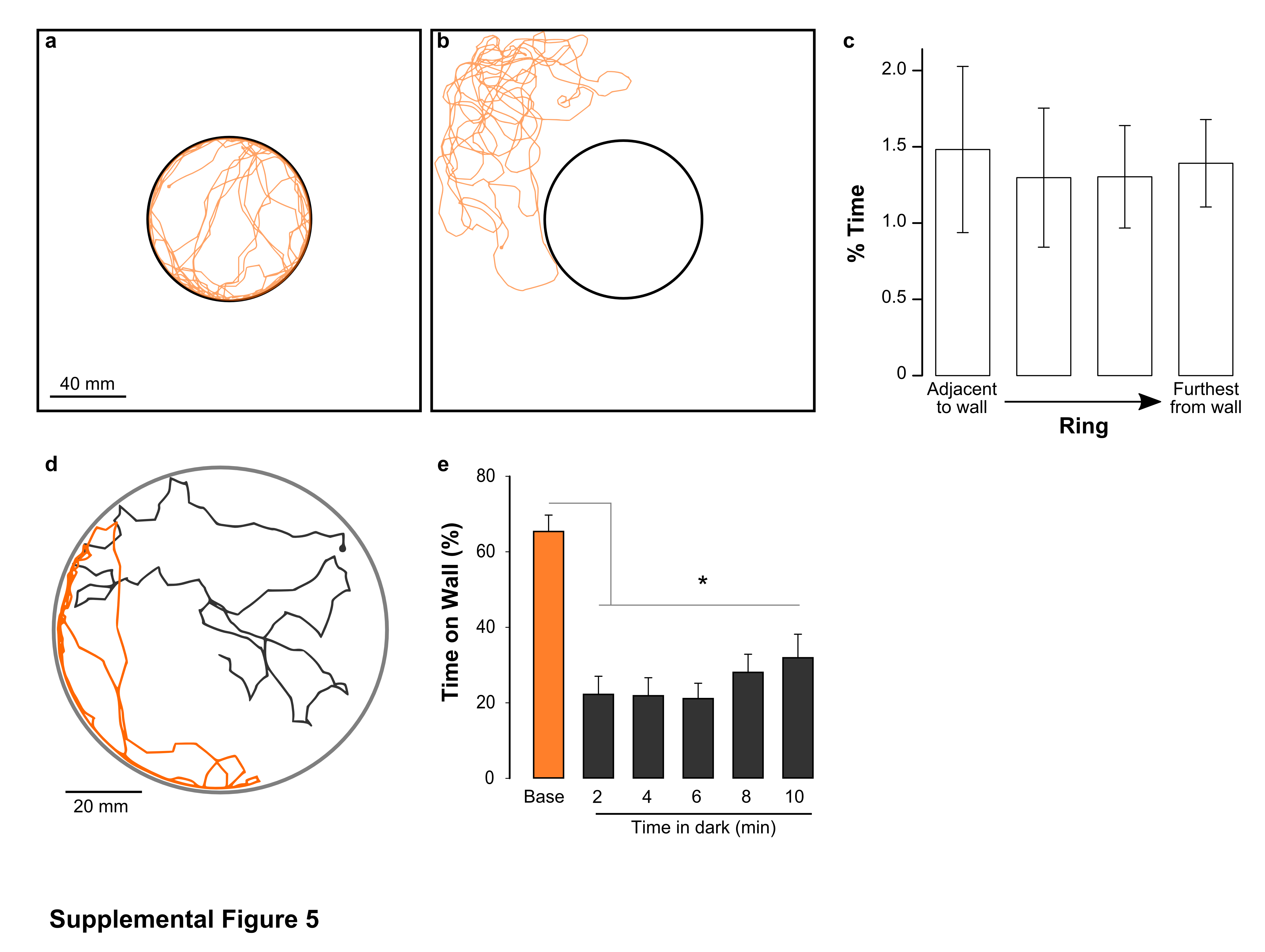

Supplement: Additional file 7: Figure S5. — Wall-avoidance behavior after loss of illumination. Illustrative path trajectories for larvae over 10 min, inside a transparent interior barrier (diameter 85 mm) within the recording chamber (a) or outside the barrier (b) under baseline conditions. (c) Time spent within concentric rings of equal area (1000 mm2) progressively further from the interior barrier during 10 min recording as in (b). Differences between time spent in the four rings are not significant (repeated measures ANOVA F3,39 = 0.19, P = 0.90), indicating that larvae do not show preferential swimming in proximity to a convex wall; N = 14 larvae. (d) Representative 2 min trajectories for larva inside the interior barrier during baseline (orange), or 8 min after loss of illumination (black). (e) Quantification of (d) – proportion of time spent by larvae within 3 mm of the wall of the chamber during baseline and at the indicated time-points after loss of illumination; N = 15. * P < 0.05. (TIF 1499 kb) [file 12915_2016_346_MOESM7_ESM.tif]

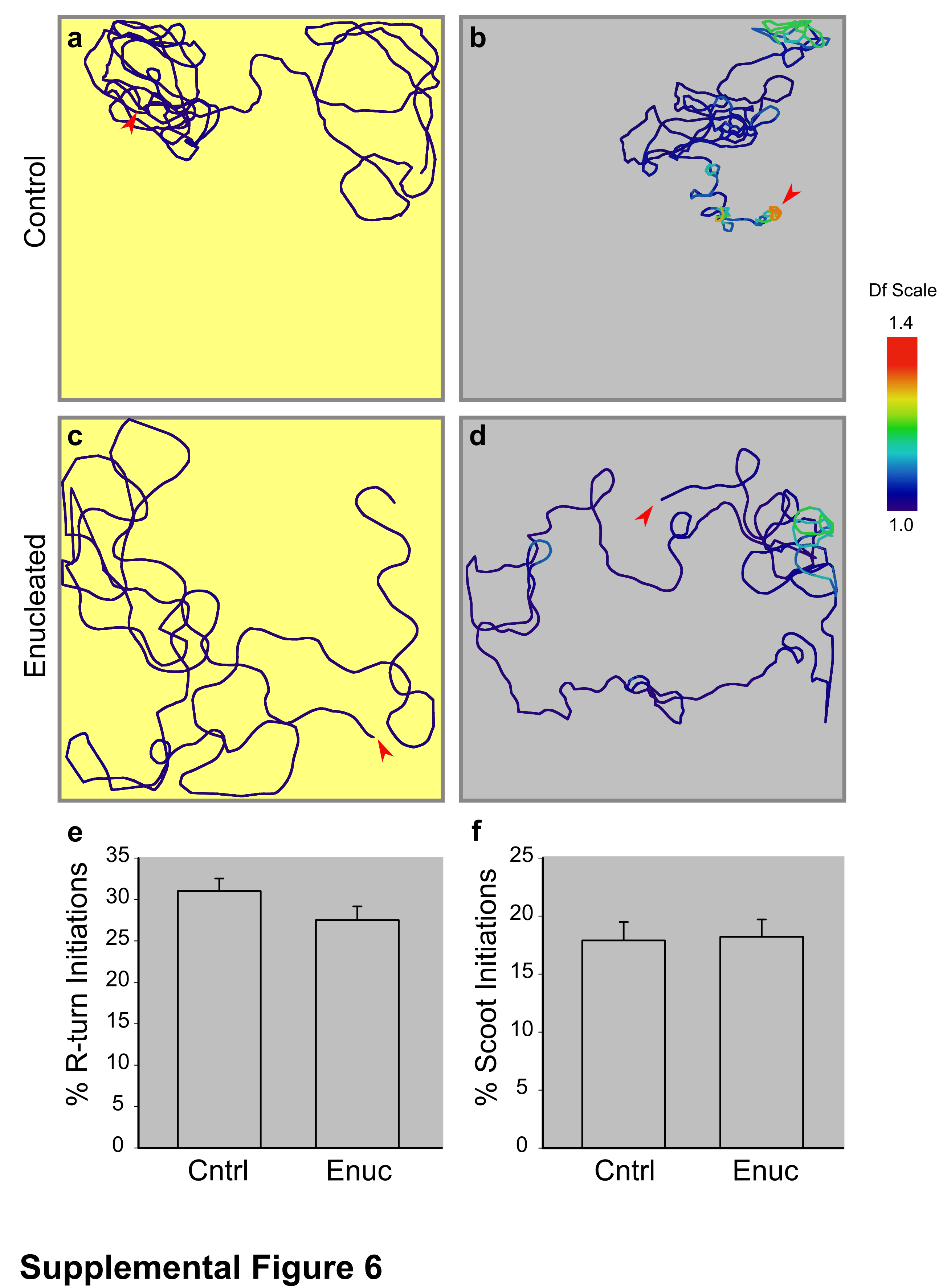

Supplement: Additional file 8: Figure S6. — Enucleated larva path trajectories. Representative 10 min trajectory for a control (a, b) and an enucleated larva (c, d) during baseline full-field illumination (a, c) and after loss of illumination (b, d). Arrow indicates starting position of larva at the beginning of the recording. Chamber: 200 × 200 mm. Color scale represents fractal dimension along path trajectory. (e–f) R-turn and slow-swim initiations were not significantly different between control and enucleated larvae after loss of illumination, demonstrating that enucleation did not broadly perturb behavior (independent sample t-test: R-turn t(121) = 1.5, P = 0.129; Scoot t(121) = 0.139, P = 0.889). (TIF 1543 kb) [file 12915_2016_346_MOESM8_ESM.tif]

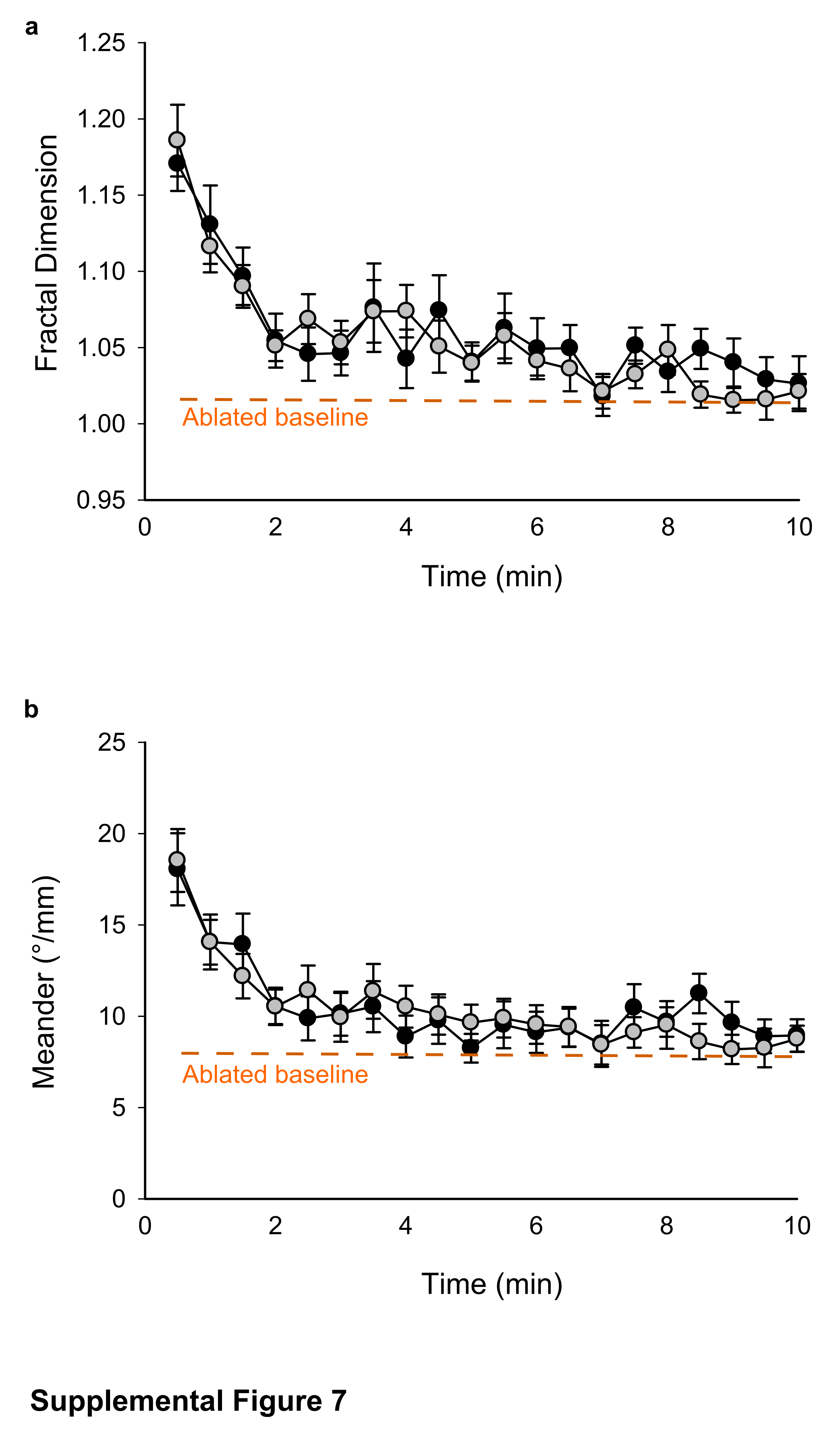

Supplement: Additional file 9: Figure S7. — Response of pineal ablated larvae to loss of illumination. (a) Fractal dimension and (b) meander after loss of illumination in pineal ablated y227Tg larvae (grey, N = 31) and metronidazole treated non-transgenic siblings (black, N = 16). Dashed line shows mean for ablated larvae under full-field illumination. (TIF 1041 kb) [file 12915_2016_346_MOESM9_ESM.tif]

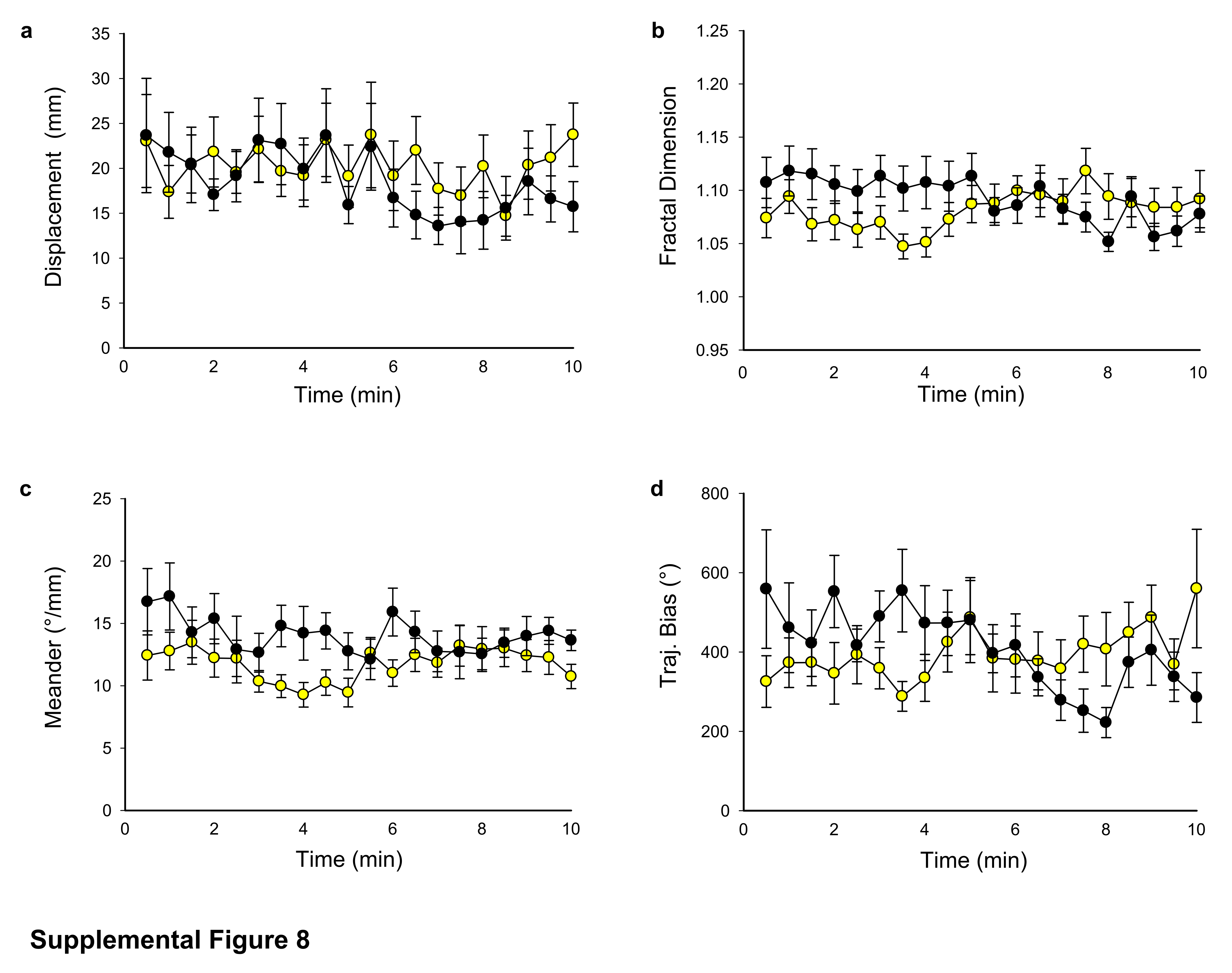

Supplement: Additional file 10: Figure S8. — Response of enucleated otpa mutant larvae to loss of illumination. Displacement (a), path complexity (fractal dimension, b), rate of re-orientation (meander, c), and trajectory bias (d) for enucleated otpa homozygous mutant larvae, recorded for 10 min under full-field illumination (yellow) or following loss of illumination (black); N = 22. Repeated measures ANOVA showed no significant main effect of enucleation on movement parameters (fractal dimension: F1,38 = 0.292, P = 0.59; displacement: F1,40 = 0.66, P = 0.42; meander: F1,38 = 2.28, P = 0.14; traj. bias: F1,38 = 0.087, P = 0.77). (TIF 1296 kb) [file 12915_2016_346_MOESM10_ESM.tif]

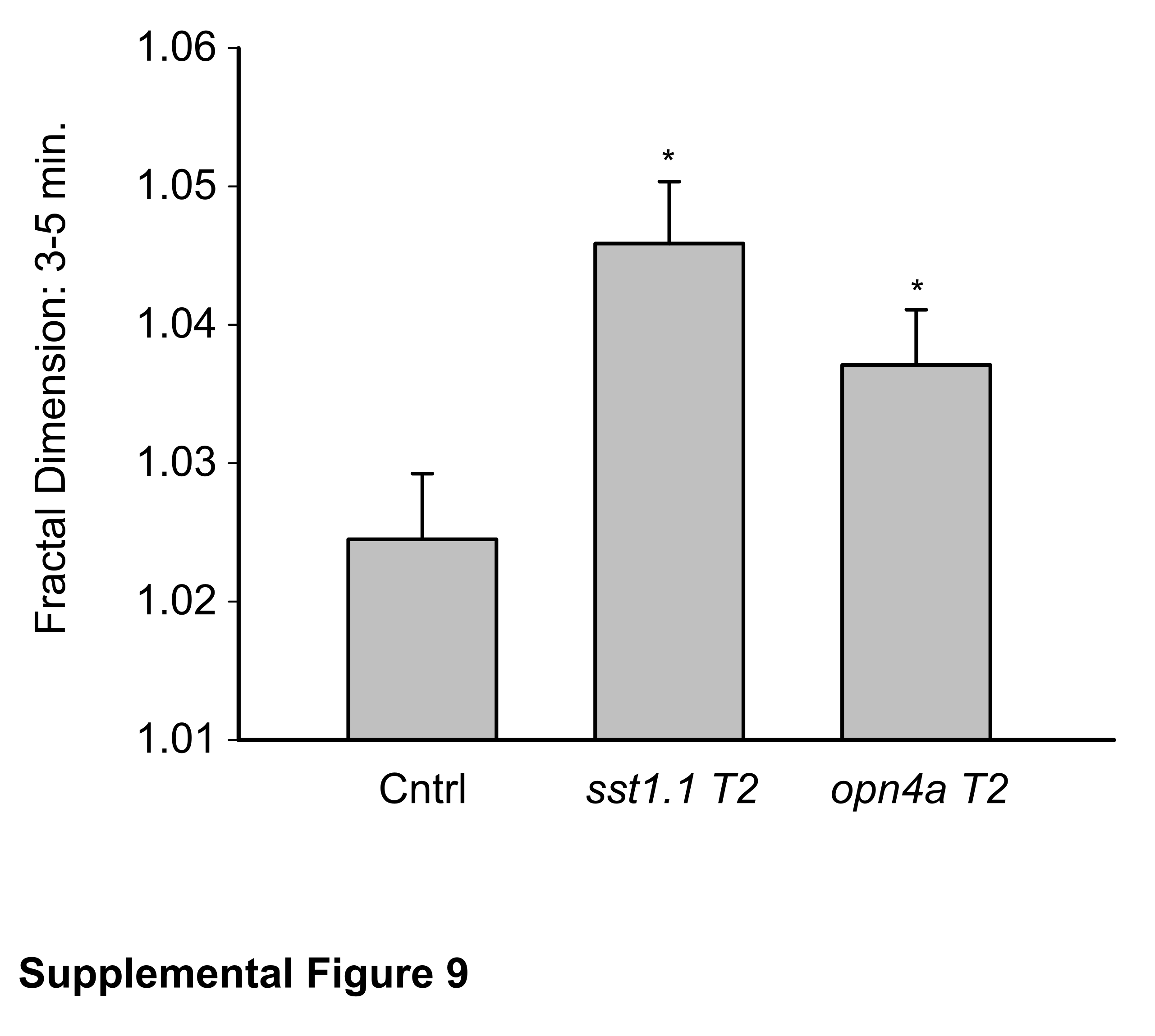

Supplement: Additional file 11: Figure S9. — Confirmation of sst1.1 and opn4a mutant phenotypes using an independent guide RNA. Path complexity for sst1.1 and opn4a mutants generated using independent sgRNAs, targeting different genomic sites than for the experiments in Fig. 7. Quantification is as for Fig. 7e. * P < 0.05 for mutants versus controls. Controls N = 26; sst1.1 T2 N = 26; opn4a T2 N = 43. (TIF 494 kb) [file 12915_2016_346_MOESM11_ESM.tif]

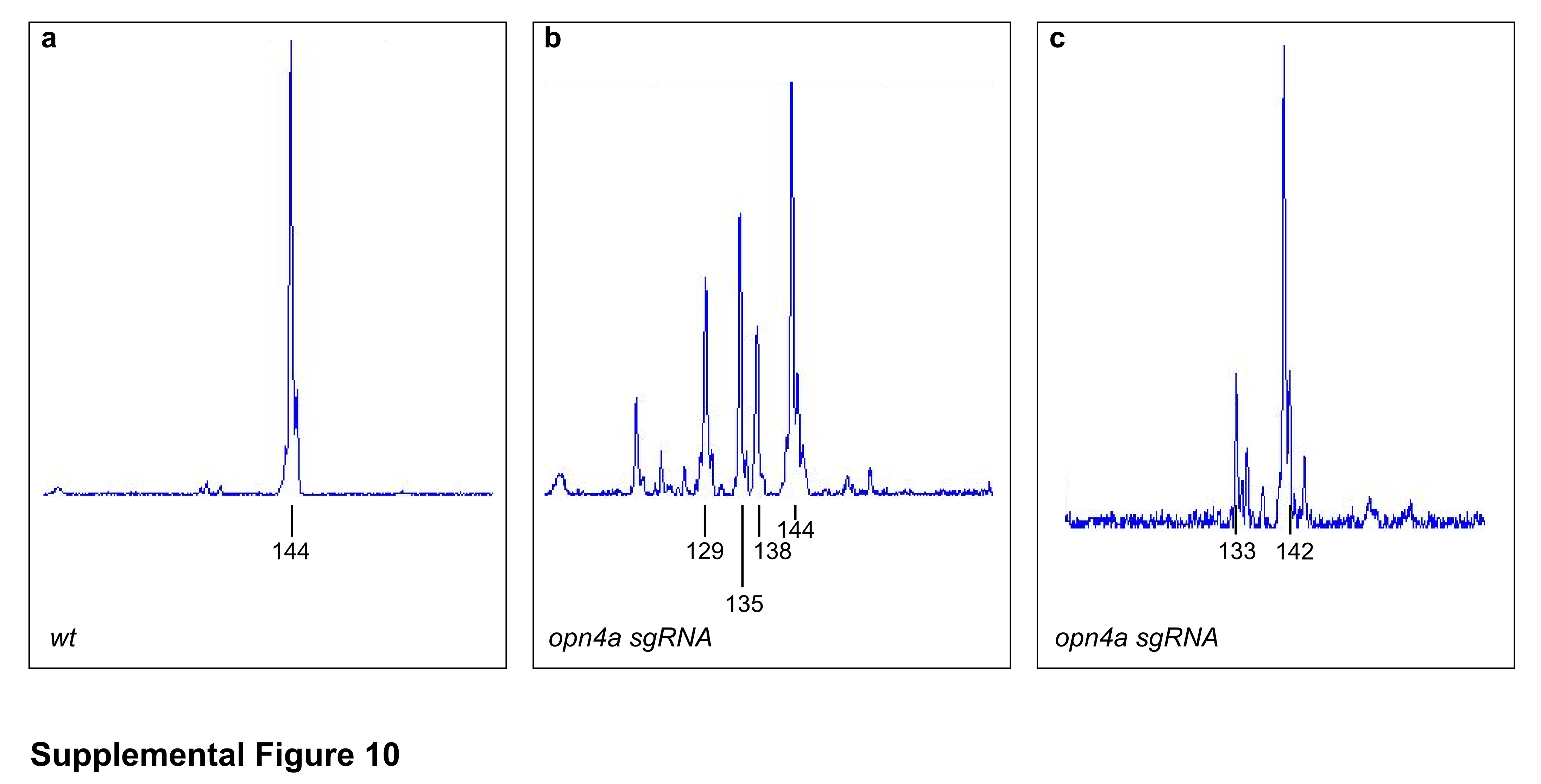

Supplement: Additional file 12: Figure S10. — Screening mutant phenotypes by CRISPR-mediated biallelic gene mutation. Representative fluorescent PCR results for amplification around the CRISPR target site in the opn4a gene in: (a) uninjected embryo showing wildtype 144 bp product from opn4a primers. (b) Embryo injected with cas9 and sgRNA against opn4a showing a residual 144 bp peak indicating incomplete mutation of opn4a. Larvae with similar genotypes were excluded from analysis. (c) Injected embryo showing complete loss of the wildtype PCR product, indicating successful biallelic mutation. Peak sizes labeled below each trace. (TIF 1371 kb) [file 12915_2016_346_MOESM12_ESM.tif]
